# Deletion of Yersinia pestis *ail* Causes Temperature-Sensitive Pleiotropic Effects, Including Cell Lysis, That Are Suppressed by Carbon Source, Cations, or Loss of Phospholipase A Activity

**DOI:** 10.1128/JB.00361-21

**Published:** 2021-10-12

**Authors:** Anna M. Kolodziejek, Carolyn J. Hovde, Gregory A. Bohach, Scott A. Minnich

**Affiliations:** a Department of Animal, Veterinary, and Food Science, University of Idaho, Moscow, Idaho, USA; b Department of Microbiology, Molecular Biology, and Biochemistry, University of Idaho, Moscow, Idaho, USA; Université de Montréal

**Keywords:** *Yersinia pestis*, Ail, lysis, phospholipid, PldA, LPS, heat shock response, membrane homeostasis, thermosensitivity

## Abstract

Maintenance of phospholipid (PL) and lipopoly- or lipooligosaccharide (LPS or LOS) asymmetry in the outer membrane (OM) of Gram-negative bacteria is essential but poorly understood. The Yersinia pestis OM Ail protein was required to maintain lipid homeostasis and cell integrity at elevated temperature (37°C). Loss of this protein had pleiotropic effects. A Y. pestis *Δail* mutant and KIM6^+^ wild type were systematically compared for (i) growth requirements at 37°C, (ii) cell structure, (iii) antibiotic and detergent sensitivity, (iv) proteins released into supernatants, (v) induction of the heat shock response, and (vi) physiological and genetic suppressors that restored the wild-type phenotype. The *Δail* mutant grew normally at 28°C but lysed at 37°C when it entered stationary phase, as shown by cell count, SDS-PAGE of cell supernatants, and electron microscopy. Immunofluorescence microscopy showed that the *Δail* mutant did not assemble Caf1 capsule. Expression of heat shock promoter *rpoE* or *rpoH* fused to a *lux* operon reporter were not induced when the *Δail* mutant was shifted from 28°C to 37°C (*P* < 0.001 and *P* < 0.01, respectively). Mutant lysis was suppressed by addition of 11 mM glucose, 22 or 44 mM glycerol, 2.5 mM Ca^2+^, or 2.5 mM Mg^2+^ to the growth medium or by a mutation in the phospholipase A gene (*pldA*::miniTn*5*, *ΔpldA*, or PldA^S164A^). A model accounting for the temperature-sensitive lysis of the *Δail* mutant and the Ail-dependent stabilization of the OM tetraacylated LOS at 37°C is presented.

**IMPORTANCE** The Gram-negative pathogen Yersinia pestis transitions between a flea vector (ambient temperature) and a mammalian host (37°C). In response to 37°C, Y. pestis modifies its outer membrane (OM) by reducing the fatty acid content in lipid A, changing the outer leaflet from being predominantly hexaacylated to being predominantly tetraacylated. It also increases the Ail concentration, so it becomes the most prominent OM protein. Both measures are needed for Y. pestis to evade the host innate immune response. Deletion of *ail* destabilizes the OM at 37°C, causing the cells to lyse. These results show that a protein is essential for maintaining lipid asymmetry and lipid homeostasis in the bacterial OM.

## INTRODUCTION

Even with exquisite knowledge of the composition and architecture of cell membranes, it is not yet understood how lipid and protein interactions maintain the integrity and stability of this essential barrier. One model of membrane analysis has been the Gram-negative bacterial outer membrane (OM), which is the primary barrier against harsh external environments and is comprised of an asymmetric lipid bilayer embedded with numerous proteins (1). Under physiological conditions, the outer leaflet of the Gram-negative OM contains lipopolysaccharide (LPS) or lipooligosaccharide (LOS). LPS consists of three components: (i) an anchoring lipid A moiety, which is a phosphorylated glucosamine dimer acylated with four to seven fatty acids depending on the Gram-negative species and environmental conditions, (ii) a highly conserved core sugar moiety, and (iii) a distal carbohydrate O-antigen, which can vary significantly even within strains of the same species. The inner leaflet of the OM and the cytoplasmic membrane contains glycerophospholipids (PL) (1). Under stress conditions (i.e., exposure to chelating agents or removal of OM proteins [OMPs]) or when LPS transport or synthesis genes are disrupted, PL from the inner leaflet are directed to the OM outer leaflet to compensate for reduced LPS concentration or disorganization. This results in PL patches within the OM outer leaflet (2, 3). When PL from the OM inner leaflet are insufficient to compensate for perturbations, the cell can traffic PL from the cytoplasmic membrane (4).

Because the presence of surface-exposed PL reduces OM barrier functions, the PL concentration must be controlled (3, 5). Phospholipase A (PldA) is an OM enzyme that catalyzes the hydrolysis of a wide range of PL substrates present in the OM outer leaflet by removal of the sn-1 and sn-2 fatty acid side chains from the glycerophosphodiester backbone of both PL and lysophospholipid (lyso-PL) (6). PldA resides in the OM as an inactive monomer under normal conditions. However, when membrane perturbations occur and PL or lyso-PL are present in the OM outer leaflet, PldA forms an active dimer with the catalytic site located in the OM outer leaflet (6). Additionally, PagP, a palmitoyltransferase, removes palmitate from PL at the sn-1 position and transfers it to lipid A. A third component for balancing OM composition is the Mla (maintenance of lipid asymmetry) system best studied in Escherichia coli. This system is an ABC transporter that regulates transport of PL between the OM outer leaflet and the cytoplasmic membrane (4, 7–9).

Yersinia pestis is a uniquely beneficial model to investigate membrane integrity, because it adapted its OM composition by genomic reduction for survival in its flea vector and to be highly virulent in mammalian hosts. Adaptations due to gene loss include a set of mutations in the O-antigen gene cluster such that lipid A is only capped by core oligiosaccharides (10). Shortened LOS is required for the type III secretion (T3SS) organelle and Ail (attachment and invasin locus) protein access to eukaryotic host cells (11, 12). The plasmid-encoded T3SS is induced at 37°C and confers a poorly understood temperature dependence for 2.5 mM Ca^2+^; loss of the pCD1 virulence plasmid or mutations in this plasmid-encoded T3SS negate this requirement for Ca^2+^. Additionally, at 37°C the lipid A of Y. pestis is predominantly tetraacylated rather than predominantly hexaacylated, as it is at 28°C. This reduced acylation at mammalian temperature obscures this pathogen-associated pattern molecule from Toll-like receptor 4 (TLR-4), allowing evasion of the innate immune response. The latter characteristic is due to a deletion of *lpxL* and a point mutation in *pagP* (13). Restoration of either gene restores hexaacylation of lipid A at 37°C with concomitant activation of TLR-4. Y. pestis with a functional *lpxL* gene is completely attenuated for virulence (14). In summary, temperature has a profound effect on acylation so that, at 28°C, LOS is hexaacylated and at 37°C it is tetraacylated.

The Ail/OmpX/PagC/Lom protein family consists of virulence-related OMPs found in E. coli (OmpX, Lom) (15, 16), Klebsiella pneumoniae (OmpK17) (17), Cronobacter sakazakii (18), Enterobacter cloacae (OmpX) (19), Salmonella enterica serovar Typhimurium (PagC, Rck) (20), Y. enterocolitica (Ail) (21), Y. pseudotuberculosis (Ail) (22), and Y. pestis Ail protein (*y1324*), also known as OmpX. Y. pestis Ail is a required virulence factor (23). Ail confers resistance to mammalian sera (11, 12, 24, 25), promotes bacterial adhesion to and invasion of host cells (11, 24, 26), and facilitates delivery of T3SS effectors to epithelial cells and leukocytes (26, 27). It also inhibits the inflammatory response and polymorphonuclear leukocyte recruitment to the lymph nodes (26, 28). Distinct from its homologues in other pathogenic yersiniae, Y. pestis Ail is expressed at both ambient (28°C) and mammalian host (37°C) temperatures (12, 21, 24, 29). It is the major protein component in the Y. pestis OM fraction, comprising an estimated 20 to 30% of the total OM proteome at 37°C, the optimal temperature for its expression (30).

Temperature regulation defines the successful life cycle of Y. pestis, as it replicates in the flea vector or mammalian host. This intricate mechanism of temperature sensing coupled to gene regulation assists the bacterium in adapting to these dramatically different conditions (31). Acquisition of a virulence-associated plasmid, pFra/pMT1, encoding murine toxin and the *caf* operon, was at the origin of Y. pestis divergence from enteropathogenic Y. pseudotuberculosis (32). The *caf* operon encodes fimbrial Caf1 protein and a chaperone-usher system allowing its export and assembly on the Y. pestis surface as a capsule (33). Together with the T3SS, the capsule suppresses phagocytosis (34, 35). Importantly, capsule assembly is temperature regulated, and the *caf1* gene is one of the most transcribed genes during mammalian infection (36).

Heat shock response is a signaling pathway responsible for temperature sensing and activation of cellular responses crucial for survival at elevated temperatures (37). Initially described to mitigate stress at elevated temperatures due to accumulation of misfolded and aggregated proteins, it was also shown to regulate other cellular components, such as PL and LPS (38, 39). In E. coli, two alternative sigma factors involved in heat shock response were described, *rpoH* and *rpoE*, responding to alterations in cytoplasm and bacterial envelope, respectively (37, 40).

Under steady-state growth conditions, RpoE is sequestered in the cell by an inner membrane-bound anti-sigma factor, RseA. Upon an increase in the rate of misfolded OM proteins in the periplasmic space, RpoE is released due to RseA cleavage by the activated DegS protease (41). Because the increase in the rate of protein misfolding is induced with increasing growth temperature (42), RpoE has a pivotal thermoregulatory role in extracytoplasmic and heat shock responses.

Subsequent to our previously reported Ail-dependent virulence phenotypes (11, 24), here we describe the unique role of Ail in maintaining lipid homeostasis in the OM at elevated temperature. A Y. pestis *Δail* mutant and KIM6^+^ wild type were systematically compared for (i) growth requirements at 37°C, (ii) cell structure, (iii) antibiotic and detergent sensitivity, (iv) analysis of proteins released into supernatants, (v) induction of the heat shock response, and (vi) physiological and genetic suppressors that restored the wild-type phenotype.

This is the first description of a thermoregulatory component to OM stability that involved Ail. Genetic and imaging data also support a recently described model of PL flow between bilayers of the OM and cytoplasmic membrane in E. coli (4). The findings contribute to the broader understanding of the dynamics between proteins and PL required for membrane stability in Gram-negative bacteria.

## RESULTS

### Y. pestis *Δail* mutant released proteins and lysed in stationary phase at 37°C.

**FIG 1:**
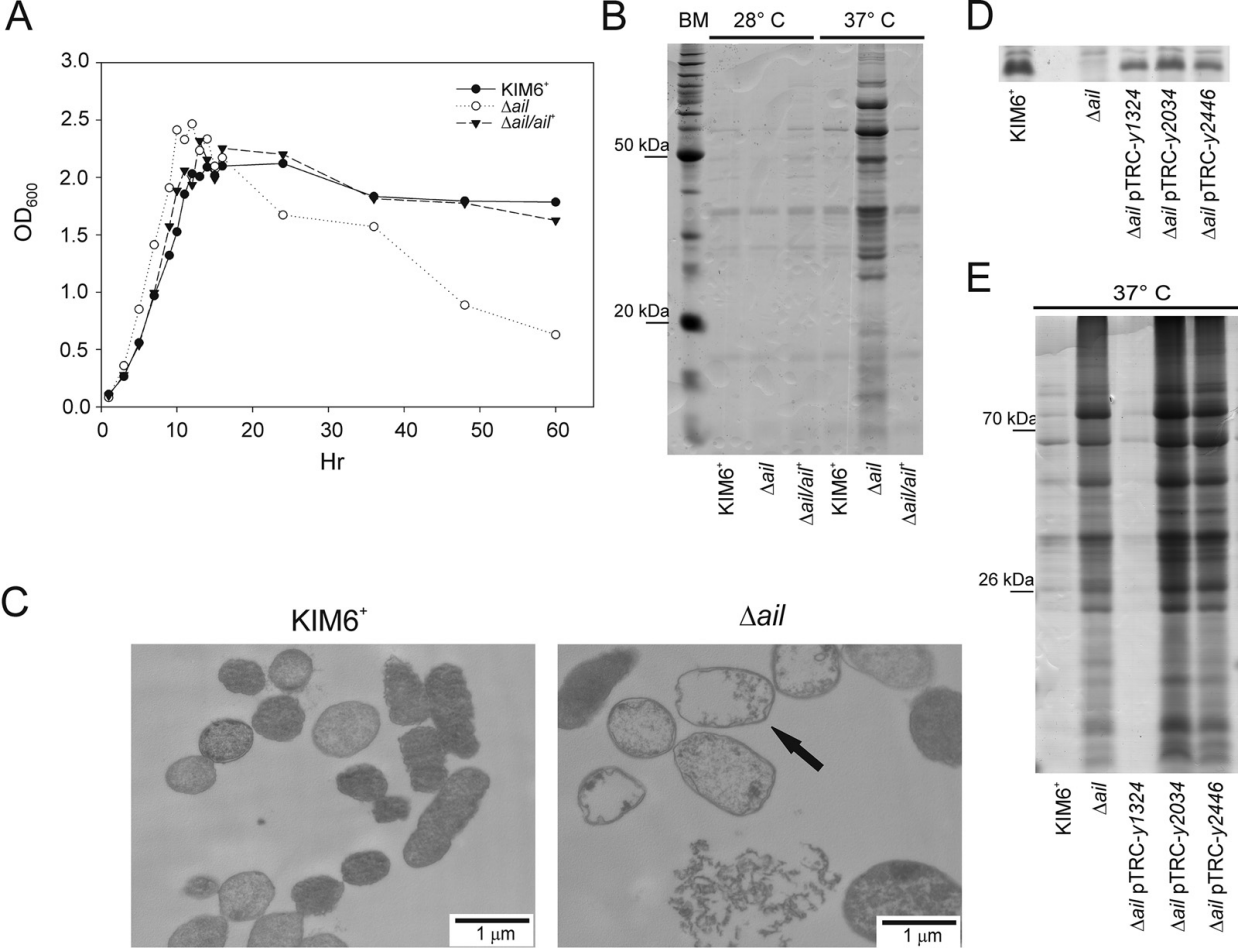
Y. pestis *Δail* mutant released proteins and lysed in stationary phase at 37°C. Y. pestis KIM6^+^ wild type, the Δ*ail* mutant, and the Δ*ail/ail^+^* complemented strain were grown with aeration to late stationary phase at 37°C or 28°C in Luria-Bertani (LB) broth. (A) Growth measured by OD_600_ shows that only the Δ*ail* mutant grown at 37°C had a significant decrease in turbidity. (B) Cell-free supernatants from cultures grown for 24 h/37°C were ethanol precipitated, separated by 12.5% SDS-PAGE, and stained with Coomassie blue (bench mark standards [BM] on left). Only the Δ*ail* mutant grown at 37°C shows increased amounts of proteins released into the medium. (C) Representative transmission electron microscopy of 24 h/37°C cultures of KIM6^+^ and the Δ*ail* mutant are shown. The Δ*ail* mutant had increased cell ghosts (arrow) and released debris. (D) Ail or Ail homologues in Y. pestis KIM6^+^ wild type, the Δ*ail* mutant, and the Δ*ail* mutant complemented with *y1324* (control), *y2034*, or *y2446*; cells were grown to mid-exponential phase in medium with 1 mM IPTG. SDS-PAGE of whole-cell lysates stained with Coomassie blue, 15- to 20-kDa range shown. (E) Proteins released from cells as indicated in panel D. Cell-free supernatants were prepared as described for panel B, and BM standard positions are indicated on the left. Only KIM6^+^ and the Δ*ail* mutant expressing *y1324* did not release increased amounts of protein into the supernatant.

Broth cultures of Y. pestis KIM6^+^ Δ*ail* mutant underwent a significant decrease in turbidity after they reached stationary phase when grown at 37°C (Fig. 1A). Culture supernatants from the stationary phase (24 h postinoculation) were collected, filtered sterilized to remove cells and particulate debris, and precipitated. Significantly more supernatant proteins were recovered from the *Δail* mutant grown at 37°C than with the wild-type parental strain or the Δ*ail*-complemented strain (Fig. 1B). No cells grown at 28°C, including the Δ*ail* mutant, released differential or significant amounts of protein into the supernatant (Fig. 1B). Plate counts showed a 10-fold decrease in viability for the Δ*ail* mutant (data not shown). Transmission electron microscopy (TEM) imaging further confirmed cell death, revealing cell lysis of *Δail* in the stationary phase (Fig. 1C). The Δ*ail* mutant phenotype had increased lysis and protein release at mammalian host (37°C) but not flea vector (28°C) temperatures.

To investigate if the *Δail* mutant lysis was specific to this protein, Ail (*y1324*) and three other Ail homologs in the Y. pestis genome (*y1682*, *y2446*, and *y2034*) were overexpressed in *trans* under an isopropyl-β-d-thiogalactopyranoside (IPTG)-inducible promoter in the *Δail* mutant as described previously by Bartra et al. (12) and tested for lysis. All constructs efficiently express the proteins in similar quantities (Fig. 1D) (12). Of the four proteins, only complementation with Ail (*y1324*) inhibited lysis of the *Δail* mutant (Fig. 1E). All cultures expressing Ail homologs lost turbidity after 72 h of growth, except for the *Δail* pTRC-*y1682* strain, which grew slowly and had reduced turbidity after 6 days (data not shown). Because of this difference in growth rate, the *Δail* pTRC-*y1682* strain was not included in the protein release assay (Fig. 1E). Interestingly, expression of *y2446* restored autoaggregation, a phenotype associated with Ail, but did not rescue cells from lysis (data not shown).

Together, these results showed that the deletion of *ail* affected Y. pestis cell stability and viability at 37°C. This effect was specific to the loss of Ail, as overexpression of the Ail homologs did not compensate for its loss.

### Y. pestis Δ*ail* mutant grown at 37°C had membrane defects in stationary phase.

To characterize changes in membrane properties between the *Δail* mutant and KIM6^+^ wild type, resistance to various membrane-active agents were compared (Table 1). Growth in the presence of antibiotic or detergent was not inhibited. Even though the *Δail* mutant was slightly more permeable to the cationic peptide polymyxin B, the MIC values for vancomycin and novobiocin indicated that the OM barrier of the *Δail* mutant was not changed. These properties were confirmed by a standard disc diffusion assay (data not shown). Further evidence that the OM of the *Δail* mutant remained functionally intact during growth was that it was slightly more resistant to the SDS anionic detergent than the KIM6^+^ wild type (Table 1).

**TABLE 1:** Antibiotic and SDS susceptibility of Y. pestis KIM6^+^ and the Δ*ail* mutant

Compound	MIC^*a*^ (μg/ml) or IC_50_^*b*^ (μg/ml) for:	
	KIM6^+^^*c*^	*Δail* mutant^*c*^
Vancomycin	>4,000	>4,000
Novobiocin	50	100
Polymyxin B	1,000	250
SDS^*d*^	226	329

a MICs are for vancomycin, novobiocin, and polymyxin B.

b IC_50_, half-maximal inhibitory concentration, applies only to SDS.

c Y. pestis grown in LB broth at 37°C for 24 h.

d Sodium dodecyl sulphate.

**FIG 2:**
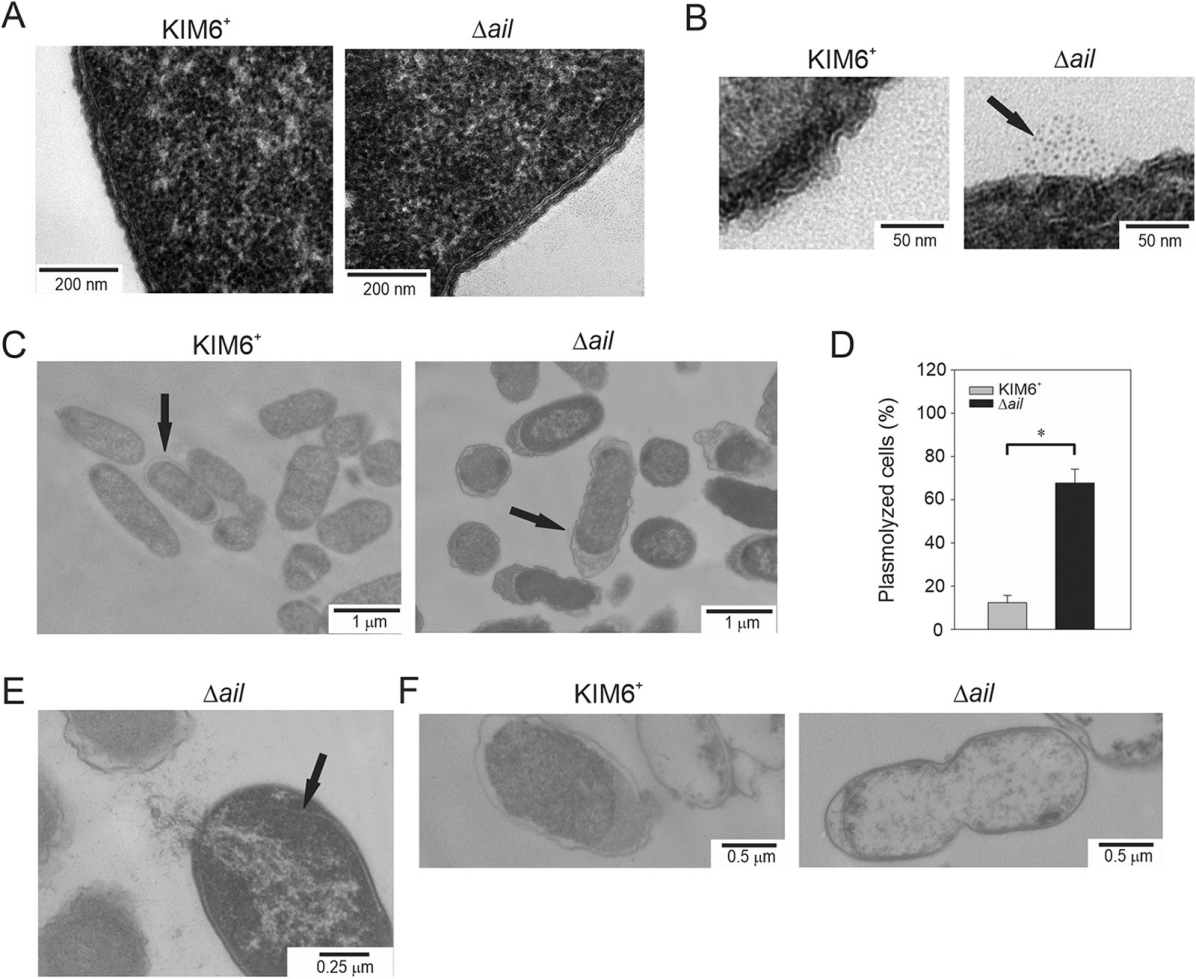
Y. pestis *Δail* mutant grown at 37°C had membrane defects in stationary phase. Y. pestis KIM6^+^ wild type and the Δ*ail* mutant were grown with aeration at 37°C in Luria-Bertani (LB) broth. Representative transmission electron micrographs of cells in the logarithmic phase (OD_600_ of 0.6) or 24 h at stationary phase are shown. In logarithmic phase, both cell types retained continuous membranes with no visible breaks or inner membrane retraction (A), but the Δ*ail* mutant released cellular matter into the supernatant (arrow) (B). In stationary phase, the Δ*ail* mutant had increased central cytoplasmic density, retracted inner membranes, enlarged periplasmic spaces (arrow) (C) and bursting cells with retracted inner membranes (arrow) (E). (F) In stationary phase, both KIM6^+^ wild type and the Δ*ail* mutant had swollen ghost cells with decreased density. (D) Percent plasmolyzed cells was quantified from 21 electron micrographs; results are means ± standard errors (SE); an asterisk indicates statistical difference (Student's *t* test, *P < *0.05).

The observations described above were corroborated by TEM imaging of bacteria collected in the mid-exponential and stationary phases of growth. Cells during logarithmic-phase growth appeared to have intact OMs. Representative images of KIM6^+^ wild type and the *Δail* mutant are shown in Fig. 2A. Nonetheless, there was release of small particles (approximately 3.5 nm in diameter) that was uniquely associated with all *Δail* mutant cells (Fig. 2B) and not observed with the KIM6^+^ wild-type cells. Imaging of the cells collected during stationary phase showed more profound differences (Fig. 2C). Quantifying this difference, 67.7% of intact *Δail* mutant cells were undergoing plasmolysis (Fig. 2D). Cells had the characteristic detached inner membranes, enlarged periplasmic spaces, and condensation of intracellular matter. Only 12.3% of the wild-type KIM6^+^ cells showed this characteristic. Among the *Δail* mutants, lysing cells were also observed, and a representative image is shown in Fig. 2E. Ghost cells, defined by two membranes with inner membrane detachment and loss of intracellular matter, were seen among both the *Δail* mutant and KIM6^+^ wild type (Fig. 2F), with fewer ghost cells in the KIM6^+^ wild-type images.

Together, results from membrane permeability assays and observations by TEM showed the membrane of the *Δail* mutant remained intact during exponential growth.

### Y. pestis Δ*ail* mutant lysis was inhibited by selected carbon metabolites and divalent metal ions.

**FIG 3:**
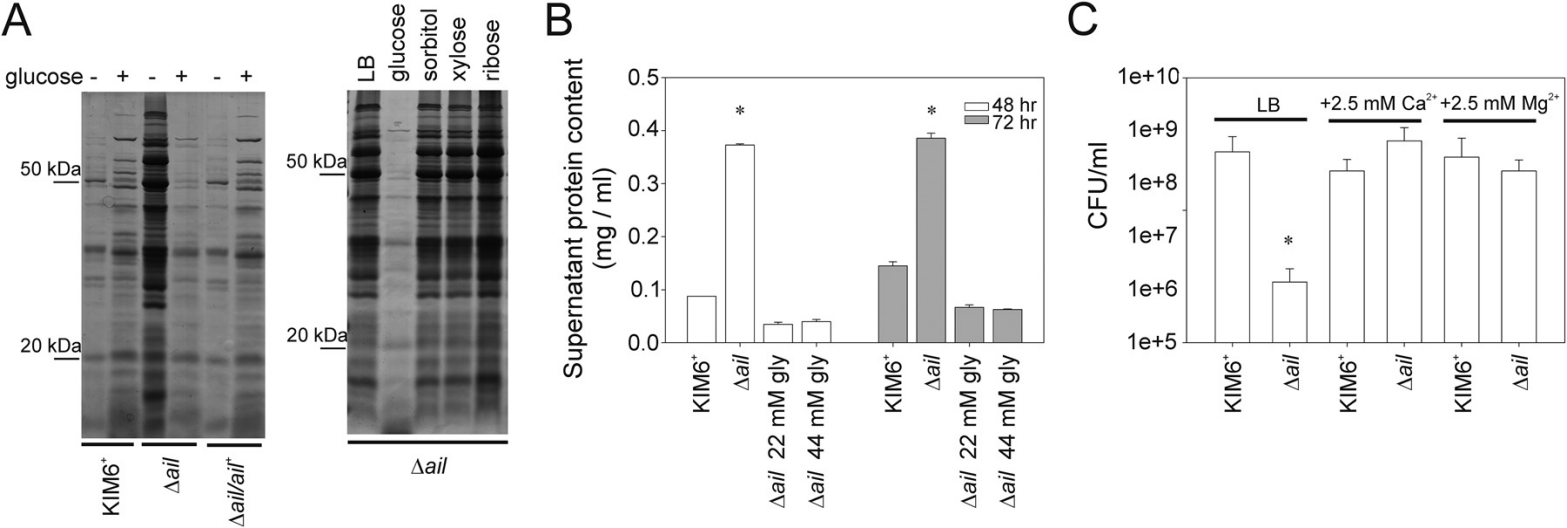
Y. pestis *Δail* mutant lysis at 37°C was prevented by glucose, glycerol, Ca^2+^, or Mg^2+^. Y. pestis KIM6^+^ wild type, the *Δail* mutant, and the *Δail/ail^+^* complemented strain were grown with aeration to late stationary phase (48 h/37°C) in Luria-Bertani (LB) broth with or without carbohydrate or divalent cations. (A) Cell-free supernatants from cultures grown with or without 11 mM glucose, sorbitol, xylose, or ribose, as indicated, were ethanol precipitated, separated by 12.5% SDS-PAGE, and stained with Coomassie blue. Protein bench mark standard positions are indicated on the left. Only growth with glucose decreased the amounts of protein released into the medium by Y. pestis *Δail* mutant. (B) The protein content of cell-free supernatants from cultures grown with or without 22 mM or 44 mM glycerol was quantified by Bradford assays. Both glycerol concentrations decreased the amounts of protein released into the medium by Y. pestis *Δail* mutant. (C) After growth in LB with or without CaCl_2_ or MgCl_2_ (2.5 mM), numbers of CFU per milliliter were determined by plate count. Growth with either divalent cation prevented Y. pestis *Δail* mutant lysis. Protein quantification and number of CFU are means ± SE from two assays performed in duplicate on separate days; asterisks indicate statistical difference (ANOVA, *P* < 0.05).

Because the *Δail* mutant replicated efficiently and reached maximal growth similar to that of the KIM6^+^ wild type and lysis was not observed until stationary phase, addition of carbon substrates or salts to the medium was tested. Glucose (11 mM) or glycerol (22 mM or 44 mM) inhibited lysis, as indicated by decreases in protein release to the supernatant (Fig. 3A and B). Even after prolonged incubation, the ***Δ****ail* mutant maintained turbidity similar to that of the KIM6^+^ wild-type cultures (data not shown). Other carbon substrate additions either failed to inhibit lysis (11 mM sorbitol or 11 mM xylose) or slightly promoted lysis (11 mM ribose), demonstrating that inhibition of *Δail* lysis was specific to glucose and glycerol (Fig. 3A).

All pathogenic Y. pestis strains require 2.5 mM Ca^2+^ at 37°C due to the virulence plasmid pCD1, and loss of the plasmid negates this calcium dependence. Interestingly, the *Δail* mutant in the calcium-dependent KIM5 (pCD1^+^) background did not lyse (data not shown). Because of this observation, even though KIM6^+^ (pCD1^−^) does not require Ca^2+^, the addition of cations was tested. In KIM6^+^, the *Δail* mutant grew similarly to KIM6^+^ wild type when either Ca^2+^ or Mg^2+^ (2.5 mM) was added to the medium (Fig. 3C).

Together, these results showed that destabilization of the *Δail* membrane can be relieved by the addition of glucose, glycerol, Ca^2+^, or Mg^2+^ to the medium.

### Analysis of proteins released by Y. pestis Δ*ail* mutant during early stationary growth phase at 37°C further defined the phenotype as lacking Caf1 capsule.

Tandem mass spectrometry (MS/MS) analysis of the supernatant from the *Δail* mutant identified catabolic and anabolic enzymes, heat shock and stringent response components, and ribosomal proteins, indicating generalized cell lysis (Table 2). Specifically lacking were phage, pesticin, and Caf1 capsule proteins. Bacterial cell lysis can result from prophage or bacteriocin induction (43–45), and the MS/MS data indicated that these processes were not involved in lysis of the Y. pestis *Δail* mutant. Consistent with this interpretation was that phage particles were not observed in TEM images (Fig. 1C and 2 and data not shown), and plaques were not generated from these supernatants on other Y. pestis, Y. enterocolitica, or Y. pseudotuberculosis strains (data not shown). To confirm that pesticin was not involved in *Δail* mutant lysis, a deletion of *pst* was engineered, and there was no difference (*P* = 0.53, Student's *t* test) in lysis between the *Δail* (1.9 × 10^7^ ± 0.6 × 10^7^ CFU/ml) and the *Δail Δpst* (2.9 × 10^7^ ± 0.9 × 10^7^ CFU/ml) double deletion mutants.

**TABLE 2:** Supernatant proteins released by Y. pestis KIM6^+^ Δ*ail* mutant during early stationary growth phase at 37°C

Gene ID	ORF	Product	Function	No. of peptides^*a*^	Protein coverage^*b*^ (%)
*y0870*	*katG*	Catalase peroxidase	Protection responses: detoxification	12	24.9
*y1004*	*tauA*	Lipoprotein	ABC-type nitrate/sulfonate/bicarbonate transport systems	4	25.6
*y1098*	*caf1M*	F1 chaperone protein	Chaperones	3	18.2
*y3165*	*ptr*	Protease III precursor	Degradation of proteins, peptides	3	5.2
*y3140*	*dapD*	2, 3, 4, 5-Tetrahydropyridine-2,6-dicarboxylate N-succinyltransferase	Amino acid biosynthesis: lysine	3	13.5
*y2629*	*gnd*	6-Phosphogluconate dehydrogenase	Energy metabolism, carbon: oxidative branch, pentose pathway	3	9.5
*y1990*	*tpx*	Thiol peroxidase	Protection responses: detoxification	3	31.1
*y1968*	*gst*	Glutathionine S transferase	Biosynthesis of cofactors, carriers: thioredoxin, glutaredoxin, glutathione	3	13.4
*y1489*	*cysK*	Cysteine synthase A	Amino acid biosynthesis: cysteine	3	17.4
*y3986*	*tufB*	Elongation factor Tu	Proteins–translation and modification	2	9.1
*y3310*	*tktA*	Transketolase	Central intermediary metabolism: nonoxidative branch, pentose pathway	2	4.1
*y2756*	*pepN*	Aminopeptidase N	Degradation of proteins, peptides	2	2.9
*y0814*	*eno*	Phosphopyruvate hydratase	Energy metabolism, carbon: glycolysis	2	6.7
*y0769*	*lpdA*	Dihydrolipoamide dehydrogenase	Energy metabolism, carbon: E3 component of pyruvate and 2-oxoglutarate dehydrogenase complex	2	6.7
*y0609*	*mopA*	Chaperonin GroEL	Chaperones	2	7.7
*y0464*	*fadB*	Multifunctional fatty acid oxidation complex subunit alpha	Degradation of small molecules; fatty acids	2	4.5
*y0444*	*udp*	Uridine phosphorylase	Salvage of nucleosides and nucleotides	2	14.2
*y0360*	*trxA*	Thioredoxin 1	Biosynthesis of cofactors; carriers: thioredoxin, glutaredoxin, glutathione	2	23.1
*y1069*	*ymt*	Murine toxin	Lipid metabolism	1	3.4
*y4135*	*atpD*	F0F1 ATP synthase subunit beta	ATP-proton motive force interconversion	1	3.9
*y4080*	*sodA*	Superoxide dismutase	Protection responses: detoxification	1	6.8
*y4004*	*rplF*	50S ribosomal protein L6	Structural component; ribosomal proteins–synthesis, modification	1	7.9
*y3985*	*fusA*	Elongation factor G	Proteins–translation and modification	1	2.0
*y3977*	*fkpA*	FKBP-type peptidyl-prolyl cis-trans isomerase	Proteins–translation and modification	1	4.7
*y3938*	*rpe*	Ribulose-phosphate 3-epimerase	Central intermediary metabolism: nonoxidative branch, pentose pathway	1	6.1
*y3855*	*prlC*	Oligopeptidase A	Degradation of proteins, peptides	1	1.5
*y3712*	*talB*	Transaldolase B	Central intermediary metabolism: nonoxidative branch, pentose pathway	1	3.5
*y3308*	*pgk*	Phosphoglycerate kinase	Energy metabolism, carbon: glycolysis	1	4.4
*y3135*	*tsf*	Elongation factor Ts	Proteins–translation and modification	1	6.0
*y3073*	*grpE*	Heat shock protein GrpE	Posttranslational modification, protein turnover, chaperones	1	7.3
*y3069*	*sdhA*	Succinate dehydrogenase flavoprotein subunit	Energy metabolism, carbon: TCA cycle	1	2.4
*y2855*	*grxA*	Glutaredoxin 1	Carrier; biosynthesis of cofactors, carriers: thioredoxin, glutaredoxin, glutathione	1	12.6
*y2735*	*ompA*	Outer membrane protein A	Outer membrane constituents	1	3.6
*y2266*	*argS*	Arginyl-tRNA synthetase	Aminoacyl-tRNA synthetases, tRNA modification	1	2.3
*y2246*	*pykA*	Pyruvate kinase	Energy metabolism, carbon: glycolysis	1	2.5
*y2165*	*gapA*	Glyceraldehydes-3-phosphate dehydrogenase	Energy metabolism, carbon: glycolysis	1	4.2
*y2063*	*acnA*	Aconitate hydratase	Energy metabolism, carbon: TCA cycle	1	1.5
*y1953*		Hypothetical protein	Unknown; belongs to glutaredoxin (GRX) family	1	10.7
*y1802*	*icdA*	Isocitrate dehydrogenase	Energy metabolism, carbon: TCA cycle	1	3.5
*y1507*		Putative aminotransferase	Amino acid biosynthesis: alanine	1	3.9
*y1485*	*crr*	Glucose specific PTS system component	Transport of small molecules; carbohydrates, organic acids, alcohols	1	8.3
*y1027*	*clpP*	ATP dependent Clp protease proteolytic subunit	Degradation of proteins, peptides	1	9.2
*y1001*	*ribH*	Riboflavin synthase subunit beta	Biosynthesis of cofactors, carriers: riboflavin	1	12.2
*y0988*		Putative peroxidase	Protection responses: detoxification	1	5.5
*y0960*	*pepD*	Aminoacyl-histidine dipeptidase	Degradation of proteins, peptides	1	2.9
*y0947*	*gmhA*	Phosphoheptose isomerase	Surface polysaccharides and antigens	1	13.0
*y0815*	*sodC*	Superoxide dismutase	Protection responses: detoxification	1	12.8
*y0767*	*aceE*	Pyruvate dehydrogenase subunit E1	Energy metabolism, carbon: pyruvate dehydrogenase	1	1.5
*y0668*	*mdh*	Malate dehydrogenase	Energy metabolism, carbon: TCA cycle	1	3.5
*y0635*	*purA*	Adenylosuccinate synthetase	Purine ribonucleotide biosynthesis	1	2.8
*y0510*	*acs*	Acetyl-CoA synthetase	Fatty acid and phosphatidic acid biosynthesis	1	1.8
*y0480*	*rplK*	50S ribosomal protein L11	Structural component; ribosomal proteins - synthesis, modification	1	9.9
*y0477*	*tufB*	Elongation factor Tu	Proteins - translation and modification	1	4.8
*y0392*		Hypothetical protein	Unknown; potential TIM-barrel signal transduction protein	1	7.5
*y0163*		Hypothetical protein	Unknown	1	9.2
*y0132*	*sspA*	Stringent starvation protein A	Regulator of transcription; a RNA polymerase-associated protein	1	6.6
*y0125*		Isoprenoid biosynthesis protein	Unknown; putative factor	1	12.4
*y0024*	*pgi*	Glucose-6-phosphate isomerase	Energy metabolism, carbon: glycolysis	1	2.9
*y0016*	*aceA*	Isocitrate lyase	Central intermediary metabolism: glyoxylate bypass	1	3.4

a Number of peptides identified for indicated protein.

b Peptide coverage (%) of indicated protein.

**FIG 4:**
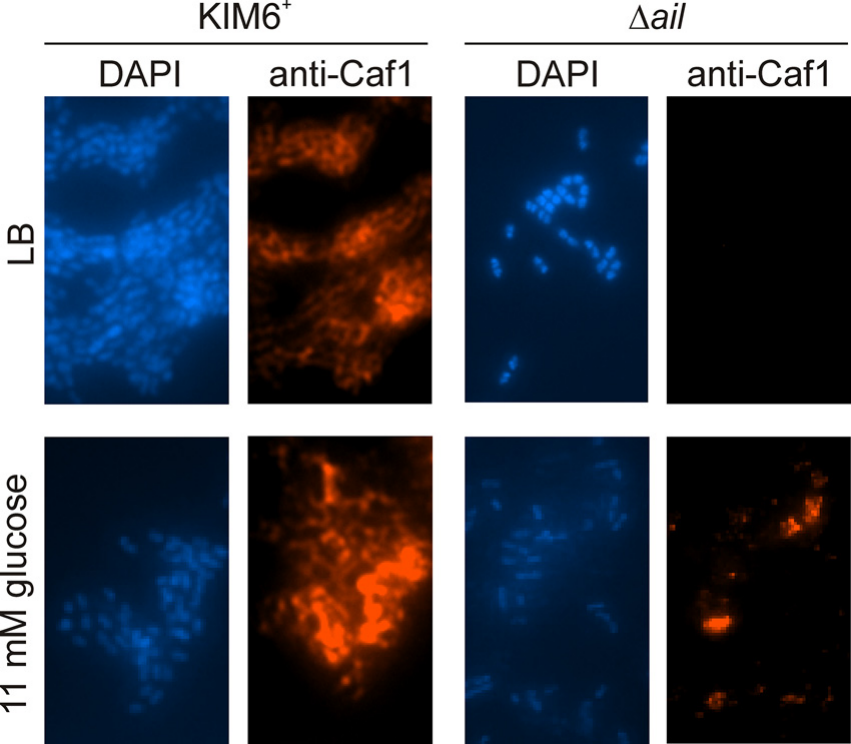
Y. pestis Δ*ail* mutant had no cell surface capsule protein (Caf1) unless glucose was added to the medium. Y. pestis KIM6^+^ wild type and the Δ*ail* mutant were grown with aeration to mid-logarithmic phase at 37°C in Luria-Bertani (LB) broth with or without 11 mM glucose. Cells were centrifuged, washed with PBS, and heat fixed on glass slides. Y. pestis cells (blue) were stained with DAPI, and Caf1 protein (red) was detected with mouse anti-Caf1 followed by goat anti-mouse Alexa Fluor 546 antibodies. Cells are at ×1,000 magnification and are representative of more than 20 microscopic fields.

The lack of Caf1 in the supernatant, normally produced and assembled on the cell surface at 37°C, was further investigated by immunofluorescence of bacterial cells. The Δ*ail* mutant had no cell surface capsule protein (Fig. 4). However, if glucose was added to the medium, it reversed this effect, just as glucose inhibited lysis (Fig. 4 and 3A).

Together, these results showed that Δ*ail* mutant lysis was not due to induction of prophage or pesticin and identified loss of capsule production as a new Δ*ail* mutant phenotype.

### Lysis of Y. pestis Δ*ail* mutant at 37°C was suppressed by mutations in the phospholipase A gene, *pldA*.

The inhibition of the *Δail* mutant lytic phenotype implied suppressor mutations could be found. A transposon screen of the *Δail* mutant employing a mini-Tn*5lacZ* revealed several independent isolates that did not exhibit cell lysis, maintained high absorbance at the optical density at 600 nm (OD_600_), and had no decrease in cell viability when incubated at 37°C (data not shown). The transposon insertions each mapped to different sites within *pldA* (phospholipase A), an enzyme required to maintain phospholipid asymmetry in the OM under stress conditions (3, 6).

**FIG 5:**
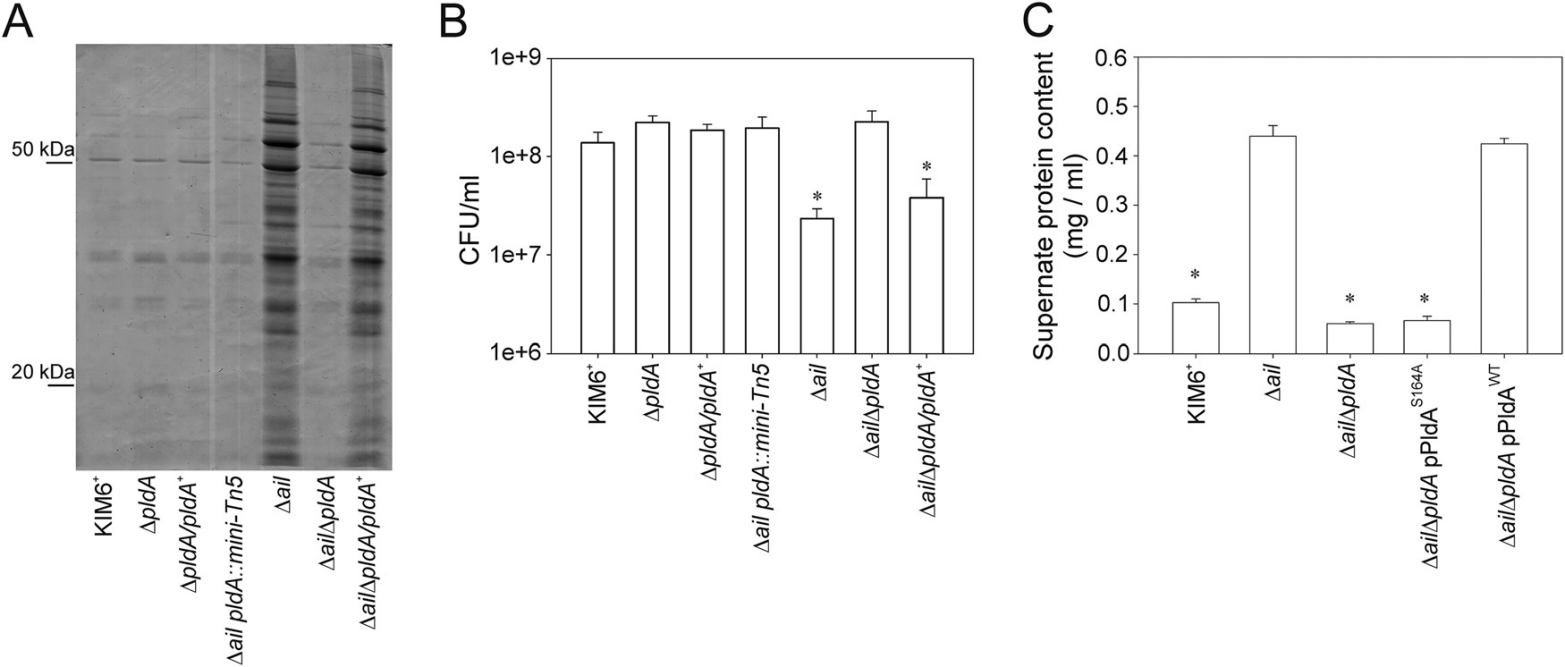
Mutations in the phospholipase A gene, *pldA*, suppressed lysis of Y. pestis *Δail* mutant at 37°C. Y. pestis KIM6^+^ wild type, the *Δail* mutant, the *ΔpldA* mutant, the catalytic site-specific PldA^S164A^ mutant, complemented strains, and the indicated double mutants were grown with aeration to late stationary phase (24 h/37°C) in Luria-Bertani (LB) broth. (A) Cell-free supernatants from cultures were ethanol precipitated, separated by 12.5% SDS-PAGE, and stained with Coomassie blue. Protein bench mark standard positions are indicated on the left. Only the wild type or strains carrying *pldA* mutations did not release increased amounts of protein into the supernatants. (B) Strains were grown as described above for 48 h and CFU numbers determined by plate count. Only the wild type or strains carrying a *pldA* mutation did not lyse. (C) The protein contents of cell-free supernatants from the catalytic site-specific PldA^S164A^ mutant and control cultures were quantified by Bradford assays. Only strains with an enzymatic phospholipase A (PldA) had increased amounts of protein released into the supernatants by Y. pestis *Δail* mutant. Results are means ± SE from two assays performed in duplicate on separate days; asterisks indicate significant difference by ANOVA (*P* < 0.05).

To confirm the role of PldA in *Δail* mutant lysis, a *pldA* deletion was constructed by site-directed mutagenesis. The Y. pestis *Δail ΔpldA* mutant lost the lytic phenotype, as determined by (i) decreased protein release into the supernatant (Fig. 5A), (ii) sustained stationary-phase viability similar to that of the KIM6^+^ wild type (Fig. 5B), and (iii) maintained absorbance at OD_600_ (data not shown). To further show that the role of PldA in lysis of the *Δail* mutant was due to enzymatic activity, the site-specific catalytic mutants PldA^S164A^ and PldA^WT^ were expressed in *trans* in the Y. pestis *Δail ΔpldA* mutant. The strains were compared for growth and lysis in stationary phase (48 h), as defined by protein release into culture supernatants. There was no difference in growth rate between the *Δail ΔpldA*, *Δail ΔpldA* (pPldA^WT^), and *Δail ΔpldA* (pPldA^S164A^) strains during logarithmic phase (data not shown). However, complementation with enzymatic PldA (pPldA^WT^) restored the lysis phenotype, unlike complementation with inactive PldA (pPldA^S164A^) (Fig. 5C).

Together, these results showed that suppressing PldA activity prevented lysis of the *Δail* mutant at 37°C and correlates with the phospholipid changes measured in this mutant.

### Downregulation of *pldA* expression occurred at 37°C and was further decreased by glucose or Ca^2+^.

**FIG 6:**
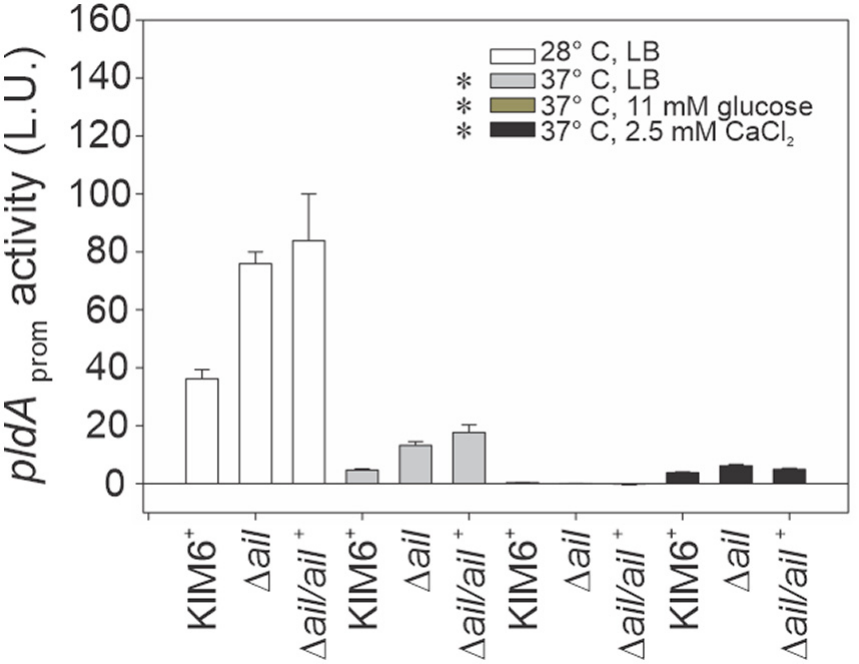
Expression of *pldA* was repressed at 37°C and further decreased by glucose or Ca^2+^. Y. pestis KIM6^+^ wild type, the *Δail* mutant, and the Δ*ail/ail^+^* complemented strain were transformed with the *lux* operon reporter under the control of the Y. pestis *pldA* promoter. Cells were grown with aeration to an OD_600_ of 1.0 at 28°C or 37°C in Luria-Bertani (LB) broth with or without 11 mM glucose or 2.5 mM CaCl_2_. Expression was measured spectrophotometrically as luminescence activity units (L.U.). Growth with glucose or Ca^2+^ repressed *pldA* expression at 37°C. Results are means ± SE from at least two assays performed in triplicate on separate days; an asterisk indicates significant difference between the growth conditions (two-way ANOVA, *P* < 0.05).

Because a deletion of *pldA* reversed the temperature-sensitive lytic phenotype of the *Δail* mutant, *pldA* regulation was investigated. The Vibrio harveyi *lux* operon transcriptional reporter system was placed under the control of the Y. pestis Kim6^+^ *pldA* promoter. The effects of temperature and glucose or Ca^2+^ on *pldA-lux* expression were assayed, analyzed with a two-way analysis of variance (ANOVA), and found to be different (Fig. 6). Based on *pldA* suppressor results (Fig. 5), the simplest prediction was that *pldA* expression would be higher at 37°C in the *Δail* mutant background than that for the *Δail* mutant at 28°C. Contrary to this expectation, the overall levels of *pldA* expression were lower at 37°C than 28°C (Fig. 6) for all strains, including the *Δail* mutant, with a 7.8-fold decrease in the KIM6^+^ wild type and 4.3-fold decrease in *Δail* mutant and *Δail/Δail^+^* complemented strain (*P* < 0.0001, one-way ANOVA, Tukey's honestly significant difference [HSD]). It is noteworthy that under these temperature conditions the *Δail/ail^+^* complemented strain had increased *pldA* expression compared to the KIM6^+^ wild type and was similar to the *Δail* mutant.

Interestingly, glucose in the medium completely repressed *pldA* expression in all strains at 37°C (*P* < 0.0001, one-way ANOVA, Tukey’s HSD) (Fig. 6). The complete inhibition of *pldA* expression and lysis (Fig. 3) by glucose may provide insights into the mechanism of its suppressive effects. Addition of Ca^2+^ to the medium also repressed *pldA* but to a lesser extent than glucose. Ca^2+^ had an average fold decrease of 2.5 in *pldA* expression in all strains (Fig. 6).

Together, these results showed that *pldA* expression was temperature, glucose, and Ca^2+^ dependent. Glucose and, to a lesser extent, Ca^2+^, significantly repressed *pldA* expression, consistent with the suppression of lysis at 37°C in the *Δail* mutant and in *Δail* mutant strains with *pldA* mutations. Lower levels of *pldA* expression at 37°C versus 28°C suggest that thermosensitive PldA-dependent lysis of the *Δail* mutant is defined by the availability of PldA substrates in the OM outer leaflet rather than the PldA concentration.

### Expression of the heat shock sigma factor promoters *rpoE* and *rpoH* was decreased in the Y. pestis *Δail* mutant at 37°C.

Lysis of the *Δail* mutant at 37°C, and not at lower temperatures, reflected its thermosensitivity. In addition, heat shock factors GroEL, PrlC, and GrpE were released into the medium at 37°C (Table 2). Thus, the expression of two sigma factors, RpoE and RpoH, that regulate the extracytoplasmic and cytoplasmic heat shock responses (46), respectively, was investigated. The Vibrio harveyi *lux* operon transcriptional reporter system was placed under the control of the Y. pestis KIM6^+^ *rpoE* or *rpoH* promoters. KIM6^+^ wild type, the Δ*ail* mutant, and Δ*ail/ail^+^* complemented strain with the *lux* constructs were grown to mid-logarithmic phase at 28°C or 37°C, and luminescence was measured.

**FIG 7:**
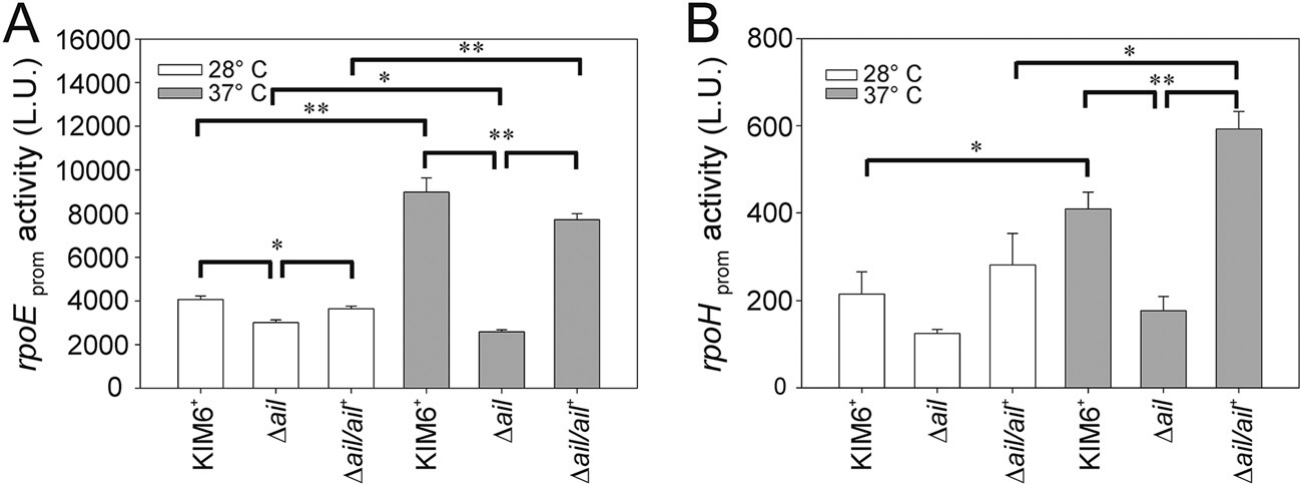
Expression of the heat shock sigma factor promoters *rpoE* and *rpoH* was decreased in the Y. pestis *Δail* mutant at 37°C. Y. pestis KIM6^+^ wild type, the Δ*ail* mutant, and the Δ*ail/ail^+^* complemented strain were grown with aeration to an OD_600_ of 1.0 at 28°C or 37°C in Luria-Bertani (LB) broth. Each strain was transformed with the *lux* operon reporter under the control of the Y. pestis *rpoE* (A) or *rpoH* (B) promoter. Expression was measured spectrophotometrically as luminescence activity units (L.U.). Results are means ± SE from at least three assays performed in triplicate on separate days; an asterisk indicates *P* < 0.05, and a double asterisk indicates *P* < 0.001 (Student's *t* test).

There was no induction of *rpoE* at 37°C above levels observed at 28°C (Fig. 7A) in the Δ*ail* mutant; expression of *rpoE* in the Δ*ail* mutant was 3-fold lower than that in the KIM6^+^ wild-type and Δ*ail*/*ail*^+^ strains during growth at elevated temperature (Student's *t* test, *P* < 0.001). Both KIM6^+^ wild-type and Δ*ail*/*ail*^+^ complemented strains increased *rpoE* expression at 37°C compared to 28°C (Student's *t* test, *P* < 0.001). In contrast, a small decrease (Student's *t* test, *P* < 0.01) in *rpoE* expression in the Δ*ail* mutant was observed at elevated temperature (Fig. 7A). Decreased levels of *rpoE* expression in the Δ*ail* mutant versus KIM6^+^ wild-type and Δ*ail*/*ail*^+^ strains were also found at 28°C, but the difference was small (average of 1.2-fold; Student's *t* test, *P* < 0.01).

A similar pattern was observed in *rpoH* regulation (Fig. 7B); there was no induction of *rpoH* at 37°C in the Δ*ail* mutant. While *rpoH* activation increased 1.8-fold in KIM6^+^ wild-type and Δ*ail*/*ail*^+^ strains at 37°C (Student's *t* test, *P* < 0.01), no statistical difference was observed between *rpoH* levels at 28°C and 37°C in the *Δail* mutant. The Δ*ail* mutant also showed lower levels of expression than KIM6^+^ wild-type and Δ*ail*/*ail*^+^ strains at 28°C. Altogether, these data indicated that deletion of *ail* suppressed the induction of two critical heat shock response sigma factors and showed Ail to be a key signaling component of the Y. pestis thermoregulatory system.

In the well-studied E. coli RpoE-induced responses, DegP is a periplasmic chaperone and protease essential for growth and viability at higher temperatures (47). The protein reduces the rate of misfolded and denatured proteins present in the periplasmic space and, thus, alleviates extracytoplasmic stress. The inability to rescue the Δ*ail* mutant from lysis by overexpression of either DegP or its protease-deficient mutant, DegP^S210A^ (data not shown), suggested that lysis was not due to compromised maturation of the OM proteome. To assess if induction of other RpoE regulon components could suppress lysis, an E. coli H198PDegS*Δ*PDZ protein was expressed in the Δ*ail* mutant. DegS is a serine endoprotease that activates RpoE by releasing it from the inner membrane in response to the presence of misfolded OMPs in periplasmic space. The mutation H198P and deletion of the PDZ domain stabilize its active form and increase its catalytic activity (41). Preliminary results showed that even though *rpoE* expression in the *Δail* mutant at 37°C increased by only 2-fold (see Fig. S1A in the supplemental material), contrary to our expectations, this strain showed enhanced lysis instead of inhibition (Fig. S1B). This DegS enhanced lysis phenotype was inhibited in the Δ*ail* Δ*pldA* background, and luminescence showed that *pldA* expression was not part of the RpoE regulon (Fig. S1B and C). These data indicated that lysis of the *Δail* mutant was regulated by components of the RpoE regulon involved in lipid homeostasis rather than protein stability.

## DISCUSSION

**FIG 8:**
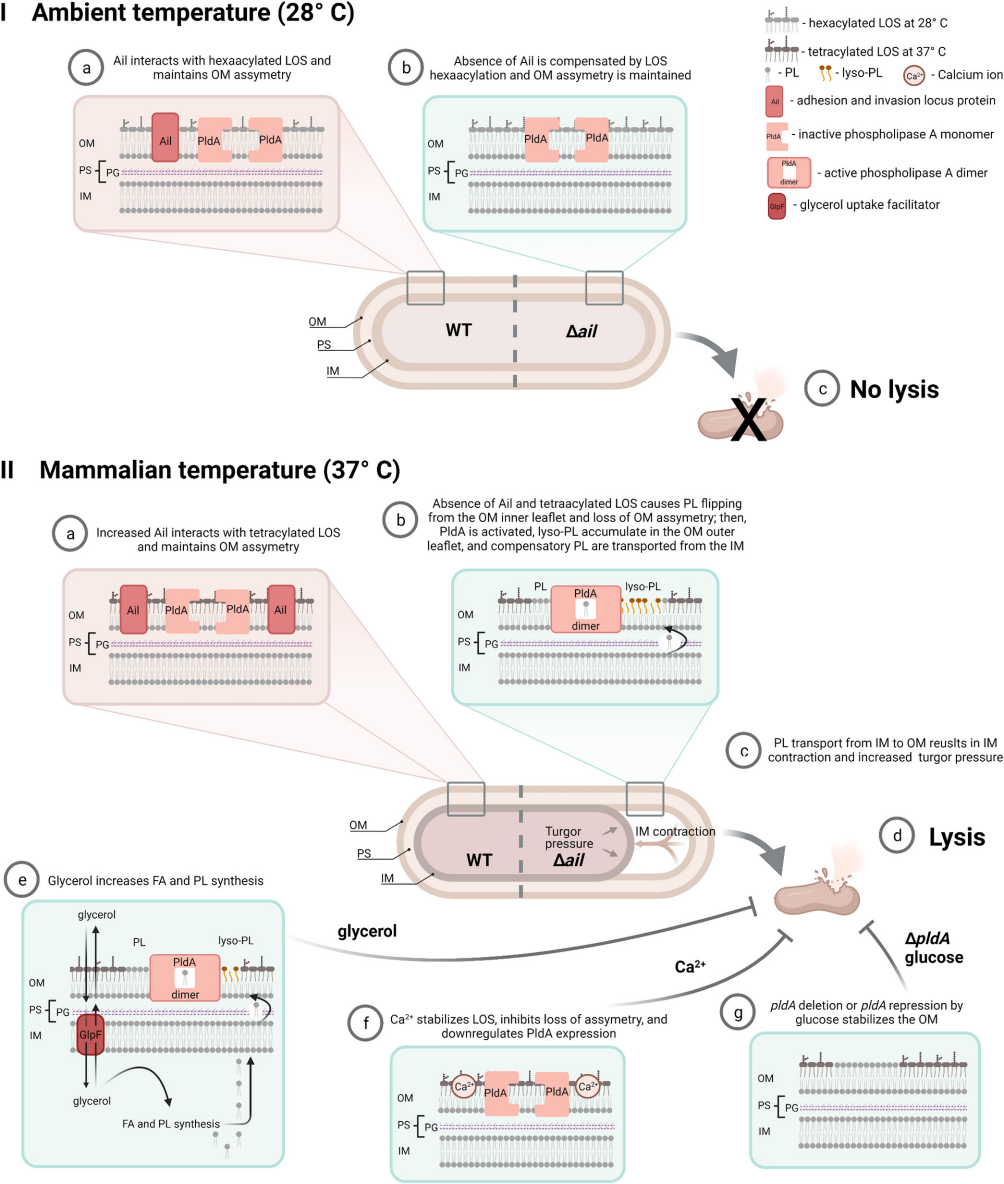
Model of temperature-dependent Ail contribution to OM stabilization. Panel I shows conditions for maintaining OM asymmetry and cell lysis prevention at ambient temperature. Panel II, a, b, and c, compares the wild type and the Ail mutant for OM disruption and cell lysis at mammalian temperature. Panel II, d, e, and f, shows conditions that suppress cell lysis. OM, outer membrane; PS, periplasmic space; PG, peptidoglycan; IM, inner membrane; LOS, lipooligosaccharide; PL, phospholipid; lyso-PL, lysophospholipid; FA, fatty acid. Figure created with BioRender.com.

The most important finding of this study was that the deletion of Y. pestis *ail*, encoding a single OMP, resulted in significant, and not previously described, pleiotropic effects. These effects included a temperature-sensitive lysis due to membrane destabilization, lack of capsule assembly, and disruption of stress response signaling associated with nutrient deprivation and temperature changes. The lytic phenotype could be suppressed physiologically by supplementation of growth media with glucose, glycerol, or cations or genetically by mutations in *pldA*. Together, these results indicate loss of a major OMP, until now primarily associated with virulence, can also have significant implications in cell signaling and growth. A model summarizing this complex system is shown in Fig. 8.

The Ail-associated lytic phenotype is temperature dependent and Ail specific. It was not recognized previously because the conditions used in studies examining Ail contributions to virulence (11) employed Y. pestis pCD1^+^ strains. This fact had several ramifications. First, all three human-pathogenic strains of *Yersinia* have the unique phenotype of being calcium dependent (2.5 mM) for growth at 37°C, conditions shown here to suppress lysis. This phenotype, referred to as the low calcium response, is pCD1 virulence plasmid mediated. Second, the virulence plasmids in pathogenic *Yersinia* are unstable at 37°C, so cultures were consistently grown at lower temperatures to ensure the plasmid was not lost. Therefore, Y. pestis pCD1*^+^ Δail* mutant strains were grown at 28°C to mid-logarithmic growth phase before conducting animal or mammalian cell culture studies at 37°C. If grown at 37°C, Y. pestis pCD1^+^ *Δail* mutants were supplemented with Ca^2+^. Therefore, the three parameters of low-temperature growth, growth to mid-logarithmic phase, and growth of cells supplemented with Ca^2+^ at 37°C were conditions that masked detection of the *Δail* lytic phenotype.

Regarding the specificity of Ail, overexpression of the other three Y. pestis Ail homologs did not compensate for the loss of Ail, indicating Ail has a specific stabilizing effect when cells are shifted to 37°C. Only complementation with *ail* stabilized the membrane. A possible role of Ail in maintaining OM homeostasis could be regulation of PldA activity via direct protein-protein interaction or sequestration of membrane-damaging products through bridging with LPS molecules, phospholipids, or both. In E. coli, recently discovered interactions between OmpC and the Mla PL transport system suggest that OMPs can specifically facilitate PL transport between the OM and inner membrane (48, 49). It will be interesting to determine if the deletion of *ail* homologs in other Gram-negative bacteria shows a similar phenotype. No reports indicating lysis of *ail* deletions for Y. enterocolitica or Y. pseudotuberculosis have been reported but may have been missed due to routine Ca^2+^ supplementation required for growth at 37°C. Thus, this phenotype may be unique to pathogenic *Yersinia* and suggests that the physicochemical properties of *Yersinia* LPS at 37°C, with decreased acylation of lipid A, are a unique contributing factor.

To detect changes in membrane permeability in the *Δail* mutant, antibiotic sensitivity was determined to (i) vancomycin, not normally active against Gram-negative bacteria due to the vancomycin-impermeable OM barrier (50); (ii) novobiocin, an indicator of the OM permeability to hydrophobic compounds (51); and (iii) polymyxin B, an antibiotic known to bind to LPS through both ionic and hydrophobic interactions (52). An increased sensitivity was found only to polymyxin B. This supports that the *Δail* mutant membrane was defective in LOS structure, PL structure, or their concentrations. Polymyxin B sensitivity of Y. pestis *ail* site-directed mutants was also reported by Singh et al. (25), and these authors mapped polymyxin B sensitivity to base cluster II residues of Ail, a region that makes direct contact with LOS. Surprisingly, the *Δail* mutant showed decreased sensitivity to SDS detergent. Less protein in the OM due to deletion of *ail* should expose more PL and a predicted increased sensitivity to anionic detergents. The fact that cells were more resistant to SDS is unexplained.

The 37°C temperature-dependent lysis phenotype of the *Δail* mutant manifests as cells enter stationary phase. Although observable release of cellular components occurred during the logarithmic phase of growth, profound morphological deterioration was not observed until stationary phase. Electron micrographs of cells at this stage show decreased cytoplasmic volume and inner membrane surface area, suggesting that cells are losing membrane faster than new PL can be synthesized. Previously reported temperature- and growth phase-dependent increased expression of proteins involved in fatty acid import (*fadL*) and catabolism (*fadI*, *fadB*, *faoA*, and *yafH*) (53, 54) suggest that Y. pestis preferentially uses lipids as an energy source at 37°C in stationary phase. Preliminary MS analysis of bacterial PL content showed differences for some PL peaks between the *Δail* mutant and wild-type cells. These differences were reduced when cells were grown in LB broth supplemented with glycerol. Whether PldA activity is specific or if glycerol leads to synthesis of stabilizing PL species needs more investigation. Genes regulating glycerol uptake (*glpF*) and metabolism (*glpK* and *glpD*) are upregulated at 37°C (54). Importantly, glucose drastically reduced expression of *pldA*, consistent with reduced cell lysis that required PldA activity. Therefore, supplementing media with glycerol or glucose, substrates that can promote lipid biosynthesis and suppress *pldA* expression, rescued cells from stationary-phase death.

Physiological suppression of lysis was also achieved by supplementing LB broth with Mg^2+^ or Ca^2+^ cations. Mg^2+^ levels are very low in LB broth (55). Ca^2+^ interacts with negatively charged LPS molecules and anionic PLs to stabilize the OM. Clifton et al. show, in a synthetic Gram-negative OM model, that removal of calcium results in a dramatic 20% mixing of LPS and PL between the inner and outer leaflet bilayers to stabilize repulsive electrophoretic forces (56). Suppression of lysis, evidenced by wild-type-level concentrations of proteins in culture supernatants, demonstrated this stabilizing effect of Ca^2+^ on OM stability. Mg^2+^ levels had an effect nearly identical to that of Ca^2+^. The expression of *pldA* was repressed by Ca^2+^. In addition, nuclear magnetic resonance (NMR) studies by Singh et al. (25) show that there is direct Ail interaction with LPS to stabilize the OM. It is likely that, here, divalent cations compensated for Ail loss, as predicted in the Clifton (56) model, by preventing PL translocation between the OM inner and outer leaflets. We concluded that excess divalent cations are necessary and sufficient to suppress the *Δail* mutant lysis phenotype.

Y. pestis synthesizes a capsule at 37°C comprised of the Caf1 protein. This protein was conspicuously missing in cell supernatant proteins released by the *Δail* mutant grown at 37°C and could not be detected by immunofluorescence with Caf1 antibody. Membrane stabilization provided by glucose supplementation to LB broth restored capsule formation at 37°C in the *Δail* mutant. This indicated loss of Ail in the OM had a disruptive effect on additional proteins. Loss of Caf1 is also consistent with the reports that a *Δail* mutant shows increased immune infiltration and phagocytosis in lymph tissue of infected rodents (26, 28). Although the lytic phenotype of the *Δail* mutant *in vitro* may alter virulence, the previously described Ail-mediated serum resistance is a major contributor to the high mortality of Y. pestis (11). Low virulence of the *Δail* mutant in a pneumonic model of plague correlates with a high potential of rat serum to kill the *Δail* cells. This trait is not observed with mouse serum, and there is no decrease in virulence in the mouse model (11). In addition, the combined blood glucose and calcium concentrations are sufficient to inhibit lysis of the *Δail* mutant *in vivo* (57).

The OM of the Gram-negative bacterium is the primary barrier against the harsh extracellular environment (1), so any disturbances in this membrane are counteracted by the cell to maintain barrier integrity. Under normal conditions, PL is excluded from the outer leaflet of the OM. However, in response to certain extracytoplasmic stress conditions, Gram-negative bacteria can accumulate PL in the outer leaflet of the OM to maintain membrane continuity (1, 2, 5). Incorporation of PL into the OM ensures OM integrity, but its selectivity and overall stability is impaired by formation of PL patches that are more permeable to small toxic molecules (1). PL in the OM activate mechanisms to regain the OM asymmetry. For example, in E. coli, the PL present in the OM outer leaflet are removed by two enzymes: the OM β-barrel PldA (3, 6) and OM β-barrel lipid A palmitoyl transferase (PagP) (58). Upon activation by mislocalized PL present in the OM outer leaflet, PldA catalyzes hydrolysis of PL or lyso-PL and removes a fatty acid residue to restore asymmetry (6). PagP acylates lipid A using a palmitate chain from an outer leaflet PL donor (59), and its expression is induced in response to the limitation of divalent cations (60). PagP activity increases heptaacylated lipid A by this palmitate addition.

The Y. pestis response to these conditions is different from that of E. coli. Acylation of lipid A is thermoregulated in Y. pestis and is the major LOS structural change between these two temperatures. Due to deletion of *lpxL* (14) and a point mutation in *pagP* (13), Y. pestis lipid A is predominantly tetraacylated at 37°C. It is interesting that *lpxL* null mutants in E. coli are conditionally lethal at temperatures above 33°C for reasons that are unclear (61). One speculation is incorporation of unsaturated palmitoleic acid in *lpxL*-null mutants is detrimental to growth at higher temperatures due to its effect on the fluidity of the membrane (62). Temperature-sensitive mutants of *lpxL* also show an abnormally high ratio of PL to protein in the OM when grown at elevated temperature. Perhaps to compensate for the deletion of *lpxL*, Y. pestis requires compensatory mutations for high-temperature growth, such as elevated expression of Ail, to stabilize the OM at 37°C. Thus, the loss of Ail in the OM may unmask the instability of tetraacylated lipid A at 37°C. Restoration of hexaacylation of Y. pestis lipid A by restoration of *lpxL* or *pagP* may compensate for the loss of Ail.

The phenotype of the Y. pestis *Δail* mutant is remarkably like the phenotype of the E. coli dominant mutation in mlaA* (maintenance of lipid asymmetry) reported by Sutterlin et al. (4). The mlaA* mutation results in increased OM permeability, blebbing in log phase, and cell lysis when cells transition to stationary growth phase. This conditional lethal mutation can be rescued by Mg^2+^ and Ca^2+^, lipid supplementation to LB broth, or by a suppressor mutation in *pldA.* Sutterlin et al. hypothesize that the mlaA* allele increases the transfer of PL from the inner to outer leaflet of the OM (a reversal of MlaA normal activity). This aberrant flow of PL into the outer leaflet activates PldA. Whether this model applies to Y. pestis is worth further examination.

While heat shock is underexplored in Y. pestis, the demonstration that deletion of Ail disrupted both RpoH and RpoE responses is significant. RpoE maintains cell envelope homeostasis by governing expression of multiple genes regulating OM protein components and genes regulating fatty acid, PL, and LPS components (63–65). The inability to rescue cells from lysis by overexpressing *rpoE*-controlled DegP or its protease-deficient mutant (both reduce misfolded proteins) is consistent with the loss of Ail primarily disrupting OM lipid asymmetry and not OM proteome stability. The overexpression of H198P DegS*Δ*PDZ, which led to constitutive *rpoE* expression in the Y. pestis *Δail* mutant and induction of genes affecting membrane stability, increased lysis rather than rescue. Similarly, in E. coli, *rpoE* is induced in stationary phase, but if overexpressed, it leads to cell lysis (66–68). The fact that DegS-enhanced lysis was suppressed in the *Δail ΔpldA* mutant also indicates that Ail and PldA are central to maintaining regulation of PL turnover and that unconstrained RpoE levels lead to lethal increases in this rate. Our data and that of others (53) show that increased expression of *rpoE* at 37°C versus 28°C inversely correlates with *pldA* expression in wild-type Y. pestis KIM6^+^. Because *pldA* was not a part of the RpoE regulon based on the *lux*-reporter fusion constructs, it will be interesting to identify indirect modes of RpoE regulation of PldA-dependent lysis. These results showed the interplay between heat shock systems, OMP, and lipids are integral to maintain OM integrity at elevated temperatures.

This work supports the structural characterization of Ail-LOS interactions in NMR models (25, 69). It also supports the broad context of lipid flow between membranes in other Gram-negative bacteria, demonstrated by the laboratories of Silhavy and Trent (4, 9). Y. pestis is an effective pathogen because of its genome reduction, but loss of *lpxL* and *pagC* have significant fitness costs if the protein content of the membrane is disturbed. Much of the work on protein-lipid interactions in the membrane has centered on the role of lipids generating a stable environment for protein function. Less attention has been paid to the converse that proteins provide a stable environment for membrane lipids. The contribution of Ail to Y. pestis membrane integrity illustrates this point.

## MATERIALS AND METHODS

### Media, strains, and primers.

Bacteria were cultured in low-salt LB broth (Luria-Bertani or lysogeny broth) (70). Congo red agar plates were used to screen for the presence of the Y. pestis pCD1 virulence plasmid (71). Antibiotics were used at the following concentrations: nalidixic acid (Nal), 50 μg ml^−1^; chloramphenicol (Cm), 30 μg ml^−1^; ampicillin (Amp), 100 μg ml^−1^; and kanamycin (Kn), 50 μg ml^−1^. Cultures were supplemented with 1 mM isopropyl-β-d-1-thiogalactopyranoside (IPTG) for induction of Ail and its homologues, DegP, DegP^S210A^, and H198PDegSΔPDZ. LB agar plates with Cm and Nal or cefsulodin-irgasan-novobiocin (CIN) *Yersinia* selective plates (BD, Franklin Lakes, NJ) with Cm were used to select single-crossover recombinants. LB agar plates with 5% sucrose and lacking NaCl were used to select double-crossover recombinants during targeted gene deletion that employed the *sacBR* locus encoding levensucrase, as described previously (11, 24). In some experiments, LB medium was supplemented with 11 mM glucose, 11 mM ribose, 11 mM xylose, 11 mM sorbitol, 22 mM or 44 mM glycerol, 2.5 mM calcium chloride, or 2.5 mM magnesium chloride. Tables 3, 4, and 5 list strains, plasmids, and primers, respectively. Only one deletion of *ail* was used with or without the Kn cassette (Y. pestis KIM6^+^ Nal^r^ Δ*ail*::*npt* or Y. pestis KIM6^+^ Nal^r^ Δ*ail*) and is referred to throughout as the Δ*ail* mutant.

**TABLE 3:** Bacteria

Strain	Relevant genotype	Source or reference
Escherichia coli		
CC1118 λ*pir*	R^−^ M^+^λ*pir*^+^	80
S17-1 λ*pir*	Δ*recA* RP4 2-Tc::*M*u-Kn::Tn*7* λ*pir*^+^ *tra*^+^ Tp^r^ Str^r^	80
TOP10	F^−^ *mcrA* Δ(*mrr-hsdRMS-mcrBC*) ϕ80*lacZ*ΔM15 Δ*lacX74 nupG recA1 araD139* Δ(*ara-leu*)*7697 galE15 galK16 rpsL*(Str^r^) *endA1*	Invitrogen
Yersinia pestis		
KIM6^+^Nal^r^	*pgm*^+^ pCD1^−^ pMT1^+^ pPCP^+^ Nal^r^	24
KIM6^+^Nal^r^ *Δail*::*npt* (previously *ΔompX*::*npt*)	*pgm*^+^ pCD1^−^ pMT1^+^ pPCP^+^ Δ*ail* Nal^r^ Kn^r^	24
KIM6^+^Nal^r^ *Δail*::*npt/ail*^+^ (previously *ΔompX*::*npt/ompX*^+^)	*pgm*^+^ pCD1^−^ pMT1^+^ pPCP^+^ *ail*^+^ with integrated pMHZ2, Nal^r^ Kn^r^ Cm^r^	24
KIM6^+^Nal^r^ *Δail*	*pgm*^+^ pCD1^−^ pMT1^+^ pPCP^+^Δ*ail* Nal^r^	This study
KIM5	*pgm* mutant pCD1^+^ pMT1^+^ pPCP^+^	S. C. Straley, University of Kentucky
KIM5 *Δail*::*npt*	*pgm* mutant pCD1^+^ pMT1^+^ pPCP^+^ Δ*ail* Kn^r^	This study
KIM5 *Δail*::*npt*/*ail*^+^	*pgm* mutant pCD1^+^ pMT1^+^ pPCP^+^ *ail*^+^ with integrated pMHZ2, Kn^r^ Cm^r^	This study
KIM6^+^ Nal^r^ *ΔailΔpst*::*npt*	*pgm*^+^ pCD1^−^ pMT1^+^ pPCP^+^ *Δail* Δ*pst* Nal^r^ Kn^r^	This study
KIM6^+^Nal^r^ *Δail pldA*::*mini-Tn5*	*pgm*^+^ pCD1^−^ pMT1^+^ pPCP^+^ Δ*ail pldA::*mini*-Tn*5*lacZ* Nal^r^ Kn^r^	This study
KIM6^+^Nal^r^ *Δail*Δ*pldA*::*npt*	*pgm*^+^ pCD1 pMT1^+^ pPCP^+^ *Δail*Δ*pldA* Nal^r^ Kn^r^	This study
KIM6^+^Nal^r^ *ΔailΔpldA*::*npt*/*pldA*^+^	*pgm*^+^ pCD1^−^ pMT1^+^ pPCP^+^*Δail pldA*^+^ with integrated pMHZ5, Nal^r^ Kn^r^ Cm^r^	This study
KIM6^+^Nal^r^ *ΔpldA*::*npt*	*pgm*^+^ pCD1^−^ pMT1^+^ pPCP^+^ *ΔpldA* Nal^r^ Kn^r^	This study
KIM6^+^Nal^r^ *ΔpldA*::*npt*/*pldA*^+^	*pgm*^+^ pCD1^−^ pMT1^+^ pPCP^+^ *pldA*^+^ with integrated pMHZ5, Nal^r^ Kn^r^ Cm^r^	This study
Enteropathogenic *Yersinia*		
Y. pseudotuberculosis	*ail^+^*, patient isolate	P. Feng, FDA
Y. enterocolitica 8081c	O:8, *ail^+^*, patient isolate	Laboratory collection

**TABLE 4:** Plasmids

Plasmid	Relevant genotype	Source or reference
pCP20	Temp-sensitive origin of replication and thermal induction of flippase synthesis; used to remove *npt* cassette from the deletion mutants, Amp^r^ Cm^r^	78
pMHZ2	Δ*ail*::*npt* gene flanked by FRT sites, used for deletion of *ail*, Kn^r^ Cm^r^	24
pEPSacB1	*mob*^+^, *pir*-dependent *ori*R6K, *sacBR*^+^ Cm^r^	79
pEPSacB1Kan	*mob*^+^, *pir*-dependent *ori*R6K, *sacBR*^+^, with Kn^r^ cassette flanked with FRT sites, Kn^r^ Cm^r^	This study
pTRC-Ail	Ail (*y1324*, OmpX) in pTrc99a, Amp^r^	12
pTRC-*y1682*	*y1682* in pTrc99a, Amp^r^	12
pTRC-*y2034*	*y2034* in pTrc99a, Amp^r^	12
pTRC-*y2446*	*y2446* in pTrc99a, Amp^r^	12
pMHZ4	pEP*sacB*1Kn containing Δ*pst*::*npt* used for deletion of pesticin, Kn^r^ Cm^r^	This study
pUTmini-Tn*5lacZ*	Suicide plasmid for mini-Tn5*lacZ* delivery, Amp^r^ Kn^r^	80
pBR322	Expression vector, Amp^r^ Tet^r^	New England Biolabs, Inc.
pMHZ5	pEP*sacB*1Kn containing Δ*pldA*::*npt* used for deletion of phospholipase A, Kn^r^ Cm^r^	This study
pUC19	Backbone vector used to clone *pldA*, Amp^r^	New England Biolabs, Inc.
pPldA^WT^	*y0396* and its 100-bp upstream regulatory fragment in pUC19, Amp^r^	This study
pPldA^S164A^	Catalytic mutant version of pPldA, Amp^r^	This study
pBAD/HisA	Used as a template for cloning of the ampicillin resistance cassette, Amp^r^	Invitrogen
pACYC177-*lux*	*luxCDABE* Kn^r^	82
pACYC177-pmrls*lux*	Promoterless pACYC177-*lux*, contains SacI fragment with Amp^r^ cassette, Kn^r^ Amp^r^	This study
pACYC177-*pldAlux*	*luxCDABE* under Y. pestis *pldA* promoter, Kn^r^	This study
pACYC177-*pldAlux2*	*luxCDABE* under Y. pestis *pldA* promoter, contains SacI fragment with ampicillin resistance cassette, Kn^r^ Amp^r^	This study
pACYC177-*rpoElux*	*luxCDABE* under Y. pestis *rpoE* promoter, Kn^r^	This study
pACYC177-*rpoElux2*	*luxCDABE* under Y. pestis *rpoE* promoter, contains SacI fragment with ampicillin resistance cassette, Kn^r^ Amp^r^	This study
pACYC177-*rpoHlux*	*luxCDABE* under Y. pestis *rpoH* promoter, Kn^r^	This study
pACYC177-*rpoHlux2*	*luxCDABE* under Y. pestis *rpoH* promoter, contains SacI fragment with ampicillin resistance cassette, Kn^r^ Amp^r^	This study
pDegP	E. coli DegP in pACYC184 under *trc* promoter, Cm^r^	R. Misra, Arizona State University
pDegP^S210A^	E. coli protease-deficient DegP in pACYC184 under *trc* promoter, Cm^r^	R. Misra, Arizona State University
pBA169	pTrc99a ΔNcoI Amp^r^	41
pRC136	E. coli H198P DegSΔPDZ-6His in pBA169, Amp^r^	41

**TABLE 5:** Primers

Application and characteristics	Primer sequence(s)^*a*^
Primers for the pMHZ2 Kn^r^ cassette with NotI and SmaI restriction sites	5′ATATATAGCGGCCGCAGATTGCAGCATTAC3′ (F),
	5′ATATACCCGGGCACAGGAACACTTAACG3′ (R)
Primers for *pst* 5′-deletion mutation with SacI and NotI restriction sites	5′TATAGAGCTCTCTTTTTGCACCAGAGCGC3′ (F),
	5′TATAAGCGGCCGCAAAAAGGGTTTAAGTTAT5′ (R)
Primers for *pst* 3′-deletion mutation with SmaI/XmaI and EcoRV restriction sites	5′ATATACCCGGGAGTTTAAAATTACTCCGGCC3′ (F),
	5′GCGGAGGATATCATGTCAGATACAATGGTA3′ (R)
Sequencing primer for identification of the Tn*5lacZ* insertion junctions	5′TTACGCTGACTTGACGGGAC3′ (F)
Primers for *pldA* 5′-deletion mutation with SacI and NotI restriction sites	5′ATATAGAGCTCATGGCGAGATTTTGGCAGA3′ (F),
	5′ATATAGCGGCCGCATAAAGTAGGAAAGGATTA3′ (R)
Primers for *pldA* 3′-deletion mutation with SmaI/XmaI and EcoRV restriction sites	5′TATTACCCGGGATGGTCGCTATAACTGGAA3′ (F),
	5′ATGCGCGATATCTTAAAGGACATCGTTCAACAT3′ (R)
Primers for *pldA* and its 100-bp upstream regulatory fragment with BamHI and HindIII restriction sites	5′ TATATGGATCCAAATCATAAAGATAAACAACA3′ (F),
	5′TATAT AAGCTTTTAAAGGACATCGTTCAACAT3′ (R)
PldA^S164A^ mutagenesis primers	5′TAACCATCAAGCCAACGGTAAA G3′ (F),
	5′AAACCAAATTCGACTTCGC3′ (R)
Sequencing primers to confirm PldA^S164A^ point mutation	5′GGCGATTAAGTTGGGTAACGC3′ (F),
	5′ATGGCACCCCAGGCTTTACAC3′ (R)
Primers for 221-bp *pldA* promoter with BamHI restriction sites	5′GCGCGCGGATCCTATGTTCTATTCTCTTC3′ (F),
	5′ATATATGGATCCAATTCCCTCACCACCCTC3′ (R)
Primers for 187-bp *rpoE* promoter with BamHI restriction sites	5′GTATAGGATCCGTTAGCCTATCTGCTCAAG3′ (F),
	5′ATATAGGATCCCCGAGGTGAACTCTCCC3′ (R)
Primers for 300-bp *rpoH* promoter with BamHI restriction sites	5′ATATGGGATCCTTATACTCTTTCCTTACC3′ (F),
	5′TATATGGATCCTTAAACCCTCTCAGT3′ (R)
Sequencing primer for *rpoE*, *rpoH*, and *pldA* promoter orientation in pACYAC117-prls*lux*	5′GGCAGACCTCAGCGCTCAAAGA3′ (F),
Primers for the Amp^r^ cassette from pBAD/HisA with SacI restriction sites	5′GCGCGGAGCTCTTTTGTTTATTTTTCTAAAT3′ (F),
	5′ATATAGAGCTCAAACTTGGTCTGACAGTTAC3′ (R)

a F, forward; R, reverse.

### Bacterial growth measurements.

Inocula from overnight cultures grown at 28°C in LB broth with aeration were diluted into fresh prewarmed LB broth, and incubation was continued at 37°C. A Beckman Coulter DU530 spectrophotometer (Beckman Instruments, Brea, CA) was used to determine culture OD_600_, with vigorous shaking before each measurement. Bacterial enumeration was done by plate count from cultures prepared as described above and grown for 48 h.

### Supernatant protein precipitation and protein quantification.

Overnight aerated cultures were grown at 28°C in LB broth with or without appropriate antibiotics. Cells were diluted 1:100 into fresh LB broth with supplements, 11 mM glucose, 11 mM xylose, 11 mM sorbitol, 11 mM ribose, 22 mM or 44 mM glycerol, or 1 mM IPTG, as indicated. Cultures were incubated for 24 h at 28°C or 37°C. Cells were removed by centrifugation (4,000 × *g*/5 min/room temperature [RT]), supernatants passed through a 0.2-μm Acrodisc filter (Pall, Corp., Port Washington, NY), and mixed with ice-cold ethanol at a 1:4 ratio. Precipitation was allowed for 2 days at 4°C. Pellets were collected by centrifugation (8,000 × *g*/10 min/4°C) and air dried. Proteins were extracted with the urea buffer (24), resolved by SDS-PAGE (72), and stained with Coomassie blue. Supernatants from cultures grown with glycerol were subjected to protein quantification by Bradford assay (ThermoScientific, Waltham, MA) according to the manufacturer’s protocol.

### TEM.

Y. pestis KIM6^+^ Nal^r^ (KIM6^+^ wild type) and the Δ*ail* mutant were incubated in LB broth with aeration at 37°C until mid-logarithmic phase (OD_600_ of 0.8) or for 24 h. Cells were harvested at mid-logarithmic phase by centrifugation (2,000 × *g*/5 min/RT), washed in Tris-EDTA buffer, and fixed as previously described (11). Cells harvested at 24 h were mixed with the fixative at a 1:1 ratio without centrifugation. The following day, cells were postfixed with 2% OsO_4_, stained with 1% tannic acid, and dehydrated (11), with the last dehydration step including acetone. Finally, samples were infiltrated with Spurr’s and acetone, embedded in resin, polymerized, sectioned (some sections were stained with 4% uranyl acetate), and viewed with a Hitachi H600 or Philips transmission electron microscope.

### Antibiotic and SDS sensitivity assays.

Vancomycin (30 μg) discs from BD (Franklin Lakes, NJ) and vancomycin, novobiocin, and polymyxin B from Sigma (St. Louis, Mo) were used. Conventional Kirby-Bauer disc plate diffusion tests (73) were used to assess antibiotic sensitivity. Standard antibiotic MICs were determined using cultures in LB broth (74). Both tests were performed at 37°C. For the SDS sensitivity assay, overnight aerated cultures were grown at 28°C in LB broth. Cells were diluted 1:100 into fresh LB broth containing serially diluted SDS (0 to 780 μg/ml) and incubated for 24 h at 37°C. Absorbances at OD_600_ were recorded and the half-maximal inhibitory concentration (IC_50_) calculated. All assays were done in duplicate or triplicate on separate days.

### MS to identify supernatant proteins.

The Δ*ail* mutant was grown overnight at 28°C in LB broth with aeration. Cells were diluted 1:100 into fresh LB broth, incubated for 24 h at 37°C, and prepared as described for the supernatant protein precipitation as described above. Proteins were resolved by SDS-PAGE (72) and stained with Coomassie blue. A 2-mm-wide vertical strip spanning each lane of gel-resolved proteins was excised, divided into five parts, destained, and trypsinized (24, 75, 76). MS/MS analysis using a Waters Nanoacquity ultraperformance liquid chromatography (UPLC) unit (Waters Corp., Milford, MA) was performed as described previously (24, 77). A ProteinLynx Global Server 2.2 and Protein Expression Informatics System software version 1.0 were used for MS/MS spectral analysis, peptide sequencing, and protein identification. MS/MS data were compared to the protein sequence databases of Y. pestis KIM from the University of Wisconsin (http://www.genome.wisc.edu/sequencing/pestis.htm) and Y. pestis CO92 from the Sanger Institute (http://www.sanger.ac.uk/Projects/Y_pestis/). Results were analyzed using Mascot software (Matrix Science, London, UK). Gene identities (ID) of protein products detected were recorded compared to the Y. pestis KIM genome.

### Phage release assay.

The Δ*ail* mutant was grown overnight at 28°C in LB broth with aeration. Cells were diluted 1:100 into fresh LB broth and incubated for 48 h at 37°C. A KIM6^+^ wild-type culture was prepared as described above, except that at 40 h, 1.5 μg/ml mitomycin C (Sigma, St. Louis, Mo) was added. After incubation, bacteria were centrifuged (4,000 × *g*/5 min/4°C) and supernatants collected and passed through a 0.2-μm Acrodisc filter (Pall, Corp., Port Washington, NY). Bacteria to agar was prepared by mixing 10 μl of overnight cultures (grown in LB broth at 28°C with aeration) of KIM6^+^ wild type, the Δ*ail* mutant, Y. pseudotuberculosis, or Y. enterocolitica 8081c, with 5 ml of tempered (45°C) 0.6% LB agar. Filtered supernatants (1, 10, or 100 μl) or medium controls with or without mitomycin C were added, mixed, and overlaid on LB agar (1.5%). Plates were incubated for 24 h at 28°C or 37°C.

### Engineering of *ail* and *pst* deletion mutations.

The *ail* deletion in Y. pestis KIM5 was made as previously described (24), except that single-crossover recombinants were counterselected on Cm CIN *Yersinia* agar plates. To make a double deletion Δ*ail* Δ*pst* mutant, the Kn^r^ (*npt*) cassette was removed using pPCP20 from Y. pestis KIM6^+^ Δ*ail*::*npt* as described by Datsenko and Wanner (78). Deletion of pesticin (*pst*, *YPPCP1.05c*) utilized a combination of methods described by Smith (79) and Datsenko and Wanner (78). The Kn^r^ cassette flanked by the flippase recognition target (FRT) sites was amplified by PCR from pKD4 (78) and cloned in pEPSacB1 (79), generating the pEPSacB1Kan plasmid. Fragments homologous to 5′ and 3′ regions of *pst* were amplified by PCR and cloned on the opposite sites of the FRT-flanked Kn^r^ cassette. The resulting construct, pMHZ4, was transformed into E. coli S17-1 *λpir* and a successful transformant was mated with the KIM6^+^ wild type and Δ*ail* mutant. Single-crossover recombinants were counterselected on LB agar with Nal and Cm as described previously (24). The merodiploid strains (*pst^+^/pst* : : *npt*) served as an isogenic precursors for selecting the *pst* : : *npt* disruptions. They were isolated on LB agar containing sucrose to select for a second crossover event while maintaining selection for the *pst*::*npt* disruption. Sucrose-resistant, Cm-sensitive colonies were tested by PCR and sequenced to confirm the deletion.

### Caf1 capsule immunostaining.

Immunostaining was done at RT; 2% bovine serum albumin in phosphate-buffered saline (PBS) was used as a blocking buffer and antibody diluent. Mouse anti-Caf1 antibody, clone YPF19 (Bio-Rad, Hercules, CA), was diluted at a ratio of 1:100, and goat anti-mouse Alexa Fluor 546 antibody (ThermoFisher Scientific, Waltham, MA) antibody was diluted at a ratio of 1:500. KIM6^+^ wild type and the Δ*ail* mutant were incubated in LB broth with or without 11 mM glucose at 37°C with aeration until mid-logarithmic phase (OD_600_ of 0.8). Cultures (1 ml) were centrifuged (4,000 × *g*/5 min/RT) and resuspended in 50 μl PBS. Cells were spread on a glass slide, air dried, and heat fixed. Samples were blocked with blocking buffer for 1 h, washed once with blocking buffer, and incubated with primary anti-Caf1 antibody for 1 h. Samples were washed thrice for 5 min and stained with secondary anti-mouse Alexa Fluor 546 antibody. After 1 h, bacteria were washed thrice with PBS for 5 min. Samples were incubated 20 min with 300 nM DAPI (4′,6′-diamidino-2-phenylindole; ThermoFisher Scientific) in PBS to stain bacterial DNA, washed briefly 5 times, and mounted with ProLong Gold (ThermoFisher Scientific; Waltham, MA) antifade reagent. Cells were visualized with a Nikon Eclipse E1000 fluorescence microscope (Tokyo, Japan) with a 100× objective. Images were acquired using a Hamamatsu Orca digital camera (Hamamatsu, Japan) and Metamorph software (Molecular Devices, San Jose, CA).

### Generalized transposon mutagenesis, library screening, and gene identification.

E. coli S17-1 λ*pir* carrying p-mini-Tn*5lacZ* (80) and the Δ*ail* mutant were mated overnight on LB agar plate at RT in several independent experiments. After conjugation, cells were resuspended in 1 ml of LB broth and plated on LB agar supplemented with Kn and Nal to select for colonies with successful Tn*5* transpositions. After a 3-day incubation at 28°C, colonies were harvested and pooled into 500 ml LB broth with Nal, Kn, and 11 mM ribose to enrich for suppressors that did not lyse. Cultures were incubated 3 days with aeration at 37°C and diluted 1:100 into fresh LB broth with antibiotics and ribose, as described above. This cycle of selection was repeated twice. Lysis suppressors were verified by turbidity comparisons with the KIM6^+^ wild type and the Δ*ail* mutant as positive and negative controls, respectively.

To identify lysis suppressor genes generated by Tn*5lacZ* insertions, total genomic DNA was purified, digested with EcoRI, cloned into the EcoRI-digested pBR322, transformed into E. coli TOP10, and selected for Kn^r^. Plasmid DNA was purified and sequenced across the transposon junction using the primer positioned upstream from the transposon 3′ end, and Y. pestis flanking DNA was identified by a standard BLAST search of the Y. pestis KIM genome.

### Genetic manipulations of *pldA*.

Deletion of *pldA* (*y0396*) was performed as described above for the *pst* deletion, with the pMHZ5 construct used for mating and Cm CIN *Yersinia* agar used for selection of the first crossover mutant. For *pldA* expression in *trans*, the *pldA* structural gene (*y0396*) and its regulatory region (100-bp upstream region) was cloned into BamHI- and HindIII-digested pUC19 vector. The point mutation (PldA^S164A^), disrupting PldA enzymatic activity, was generated using a Q5 site-directed mutagenesis kit (New England Biolabs, Inc.; Ipswich, MA). This site-directed mutagenesis was based on the findings of PldA catalytic activity in E. coli (81) and sequence homology with Y. pestis *pldA*.

### Gene reporter systems utilizing the *lux* operon.

To measure gene expression of *pldA*, *rpoE*, *rpoH*, and *lux* operon fusions were constructed. The KIM6^+^ wild-type *pldA*, *rpoE*, and *rpoH* promoters, on BamHI fragments (187 bp, 221 bp, and 300 bp, respectively), were cloned in front of the *lux* operon from Vibrio harveyi to generate reporters pACYC177- *pldAlux*, pACYC177-*rpoElux*, and pACYC177*-rpoHlux*. The correct orientation of the inserts was verified using the primers (Table 4). The SacI fragment containing the Amp^r^ cassette from pBAD/HisA (Invitrogen, Waltham, MA) was inserted into the plasmids (described above), creating pACYC177-*pldAlux*2, pACYC177-*rpoElux*2, and pACYC177-*rpoHlux*2. Y. pestis strains were transformed with these plasmids and compared to controls transformed with the promoter-less pACYC177-pmrls*lux* to measure background luminescence. Overnight cultures grown in LB Amp broth at 28°C with aeration were diluted (1:5,000) in fresh LB Amp broth with or without supplements and incubated at 28°C or 37°C to an OD_600_ of 1 or 0.8 for cultures supplemented with glucose. Luminescence was measured in white 96-well plates using the SpectraMax L (Molecular Devices, LLC, San Diego, CA) and an endpoint measurement with 1-s integration time. Data are presented as luminescence units/OD_600_ after adjustment for background luminescence.

### DegP expression.

KIM6^+^ wild-type, Δ*ail*, Δ*ail* pDegP, and Δ*ail* pDegP^S210A^ strains were grown overnight in LB broth with or without Cm at 28°C with aeration. Cells were diluted 1:100 in fresh LB broth with or without 1 mM IPTG and Cm and incubated at 37°C for 48 h, and cell densities (OD_600_) were compared.

### *rpoE* induction.

To measure induction of *rpoE* by H198PDegSΔPDZ, overnight cultures of KIM6^+^ wild-type pACYC177-*rpoElux*, Δ*ail* pACYC177-*rpoElux*, Δ*ail* pBA16 pACYC177-*rpoElux*, and Δ*ail* pRC136 pACYC177-*rpoElux* were prepared with appropriate antibiotics as described above. Cultures were diluted (1:5,000) in LB broth with 1 mM IPTG and appropriate antibiotics and incubated at 37°C to an OD_600_ of 1, and luminescence measured as described above.

To test the role of PldA in H198PDegSΔPDZ-dependent lysis, overnight cultures of KIM6^+^ wild-type, Δ*ail*, Δ*pldA*, Δ*ail* Δ*pldA*, KIM6^+^ wild-type pBA169, Δ*ail* pBA169, Δ*pldA* pBA169, Δ*ail* Δ*pldA* pBA169, KIM6^+^ wild-type pRC136, Δ*ail* pRC136, Δ*pldA* pRC136, and Δ*ail* Δ*pldA* pRC136 strains were prepared as described above. Cell lysis was determined by spotting serial dilutions on LB agar plates containing 1 mM IPTG with or without appropriate antibiotics. Plates were observed for colony clearing after incubation at 28°C or 37°C for 8 days. To determine if *pldA* was part of the RpoE regulon, cultures of the Δ*ail* mutant, Δ*ail* pBA169, and Δ*ail* pRC136, each transformed with pACYC177-*pldAlux*, were grown as described above for the gene reporter systems utilizing the *lux* operon, except that H198PDegSΔPDZ was induced with 1 mM IPTG.

### Statistical analysis.

Data were analyzed using the Student's *t* test with one-way or two-way analysis of variance (ANOVA). Effects of temperature, glucose, and Ca^2+^ on *pldA* expression was tested with the two-way ANOVA without interactions; growth at 28°C versus 37°C, growth in LB versus LB supplemented with glucose, and growth in LB versus LB supplemented with Ca^2+^ were analyzed. The absolute IC_50_ was calculated using four-parameter logistic nonlinear regression. These analyses were conducted with SigmaPlot or R software.
