# Supplementary material for: Deletion of Yersinia pestis ail Causes Temperature-Sensitive Pleiotropic Effects, Including Cell Lysis, That Are Suppressed by Carbon Source, Cations, or Loss of Phospholipase A Activity
Source: J Bacteriol. 2021 Oct 12;203(21):e00361-21. doi: 10.1128/JB.00361-21 (PMC8508112; doi:10.1128/JB.00361-21)
Supplement: Supplemental file 1 — Fig. S1. Download JB.00361-21-s0001.pdf, PDF file, 1.0 MB [file jb.00361-21-s0001.pdf]

Supplemental material

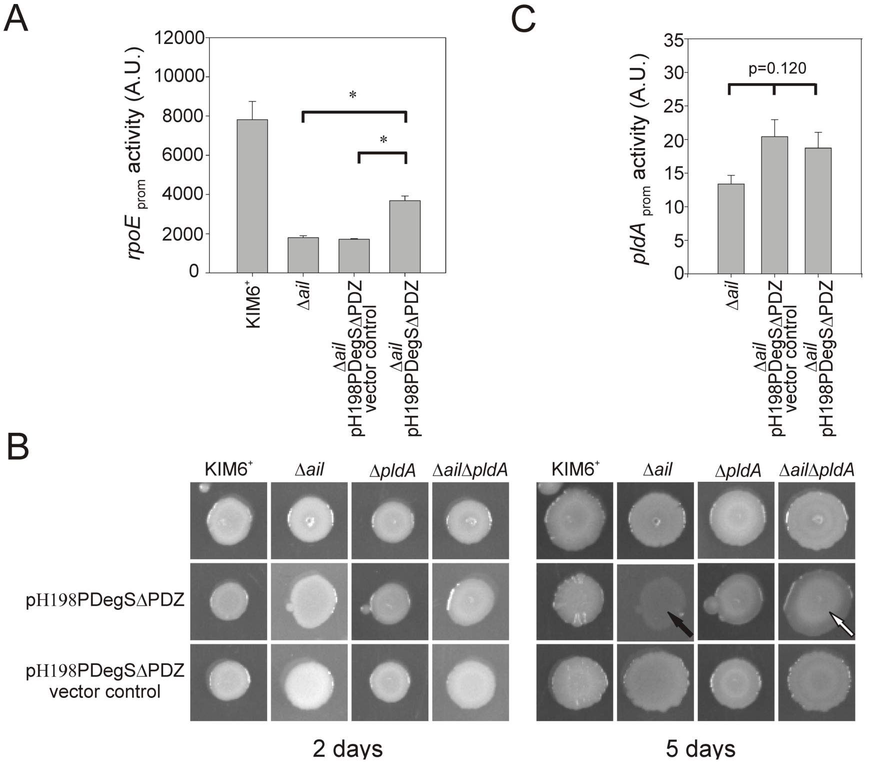

FIG S1. Lysis of the Δ*ail* mutant was regulated by components of the RpoE regulon. (A) H198PDegSΔPDZ protein increases *rpoE* expression in the Δ*ail* mutant. *Y. pestis* KIM6$^+$ wild-type, the Δ*ail* mutant, the Δ*ail* mutant expressing H198PDegSΔPDZ protein *in trans,* or its vector control were transformed with the *lux* operon reporter under control of the *Y. pestis rpoE* promoters. Strains were grown with aeration to OD_{600} = 1.0 at 37° C in Luria-Bertani (LB) broth with 1 mM IPTG. Expression was measured spectrophotometrically as luminescence activity units (L.U.). Results are means±SE from at least three assays performed in triplicate on separate days; asterisk (*) indicates $p < 0.05$ (ANOVA). **(B)** H198PDegSΔPDZ protein enhanced lysis phenotype of the Δ*ail* mutant and was inhibited in the Δ*ail*Δ*pldA* background. *Y. pestis* KIM6$^+$ wild-type, the Δ*ail* mutant, the Δ*pldA* mutant, double Δ*ail*Δ*pldA* mutant, corresponding strains expressing H198PDegSΔPDZ protein *in trans*, or their vector controls were incubated overnight at 28° C in Luria-Bertani (LB) broth with aeration and spotted on the LB agar plates amended with 1 mM IPTG. Plates were incubated at 37° C for 2 and 5 days. The Δ*ail* mutant expressing H198PDegSΔPDZ had enhanced lysis manifested by colony disappearance (black arrow). This phenotype was suppressed by deletion of *pldA* (white arrow). **(C)** *pldA* expression was not part of the RpoE regulon. The Δ*ail* mutant, the Δ*ail* mutant expressing H198PDegSΔPDZ protein *in trans,* or its vector control were transformed with the *lux* operon reporter under control of the *Y. pestis pldA* promoters. Strains were grown and *pldA* expression was measured spectrophotometrically as luminescence activity units (L.U.) as indicated in **(A).** No statistical difference (p = 0.120) in the *pldA* expression among strains was found.
